# **A systematic review of supermarket** **automated electronic sales data for population dietary surveillance**

**DOI:** 10.1093/nutrit/nuab089

**Published:** 2022-05-05

**Authors:** Victoria L Jenneson, Francesca Pontin, Darren C Greenwood, Graham P Clarke, Michelle A Morris

**Affiliations:** Leeds Institute for Data Analytics, University of Leeds, Leeds, United Kingdom; School of Geography, Faculty of Environment, University of Leeds, Leeds, United Kingdom; Leeds Institute for Data Analytics, University of Leeds, Leeds, United Kingdom; School of Geography, Faculty of Environment, University of Leeds, Leeds, United Kingdom; Leeds Institute for Data Analytics, University of Leeds, Leeds, United Kingdom; School of Medicine, Faculty of Medicine and Health, University of Leeds, Leeds, United Kingdom; School of Geography, Faculty of Environment, University of Leeds, Leeds, United Kingdom; Leeds Institute for Data Analytics, University of Leeds, Leeds, United Kingdom; School of Medicine, Faculty of Medicine and Health, University of Leeds, Leeds, United Kingdom

**Keywords:** dietary assessment, dietary surveillance, methods, supermarket, transactions

## Abstract

**Context:**

Most dietary assessment methods are limited by self-report biases, how long they take for participants to complete, and cost of time for dietitians to extract content. Electronically recorded, supermarket-obtained transactions are an objective measure of food purchases, with reduced bias and improved timeliness and scale.

**Objective:**

The use, breadth, context, and utility of electronic purchase records for dietary research is assessed and discussed in this systematic review.

**Data sources:**

Four electronic databases (MEDLINE, EMBASE, PsycINFO, Global Health) were searched. Included studies used electronically recorded supermarket transactions to investigate the diet of healthy, free-living adults.

**Data extraction:**

Searches identified 3422 articles, of which 145 full texts were retrieved and 72 met inclusion criteria. Study quality was assessed using the National Institutes of Health Quality Assessment Tool for Observational Cohort and Cross-Sectional Studies.

**Data analysis:**

Purchase records were used in observational studies, policy evaluations, and experimental designs. Nutrition outcomes included dietary patterns, nutrients, and food category sales. Transactions were linked to nutrient data from retailers, commercial data sources, and national food composition databases.

**Conclusion:**

Electronic sales data have the potential to transform dietary assessment and worldwide understanding of dietary behavior. Validation studies are warranted to understand limits to agreement and extrapolation to individual-level diets.

**Systematic Review Registration:**

*PROSPERO registration no.* CRD42018103470

## INTRODUCTION

Population dietary surveillance is important for understanding temporal changes and variation between subgroups. The data contribute to the epidemiologic understanding of diet-related diseases^1^ and enable targeting and evaluation of public health policy interventions. Current approaches to population dietary surveillance, including national surveys, rely heavily on self-reported measures of intake and food purchases. Because of their expense, surveys are restrictive in size and geographic coverage. Self-reported dietary measures are often criticized for their introduction of recall and reporting biases on the part of study participants, and possible coding errors by researchers,^2^ resulting in a tendency to underestimate intake.^3^ Moreover, it is not possible for national surveys to collect data continuously or in real time, which limits their utility.

Supermarkets dominate as the source of household food supplies in high-income countries. Thus, supermarket purchase records may offer insight into diets in high- and middle-income settings. Early work using paper cash-register receipts highlighted the feasibility of supermarket purchase data to contribute to population dietary surveillance.^4^ All this was promising, the paper-based nature of data collection limited scale and timeliness and relied on manual researcher coding.^4^^,^^5^ Recent advancements in computational storage and power preceded a movement for repurposing commercial “big data” sources^6^^,^^7^ to address public health and social science questions.^1^^,^^8^^,^^9^ Supermarket electronic transaction records, generated as a byproduct of daily activity, build upon the earlier foundations of paper-based receipt collection.^4^ However, they capture purchases rather than consumption and exclude foods eaten out of the home or purchased or obtained elsewhere. Exploration of the utility of supermarket transaction records in nutrition research, therefore, is warranted.

A previous review of both paper-based and electronically captured transaction records suggested that supermarket data could contribute to dietary research in 7 key areas; 1) dietary patterns, 2) longitudinal analysis, 3) nutrient availability, 4) validation of self-report, 5) identifying predictors of healthy food choices, 6) evaluating intervention effectiveness, and 7) exploring associations between diet and health outcomes.^10^ Electronically captured purchase data could offer benefits over paper-based methods as a more cost-effective, low-burden tool for monitoring household dietary purchases, longitudinally and at scale.^10^ However, the review emphasized that challenges related to data linkage and data sharing must be overcome. Furthermore, there is a need for robust analytical methods and to establish correction factors to account for differences between food purchases and consumption.^10^

Similarly, Bandy et al,^11^ in a recent systematic review, highlighted the utility of purchase data from households participating in commercial market-research panels as a source of dietary surveillance information. Benefits of market-research panel data include a large population, coverage of retailers, and temporal granularity and makes the data useful for evaluating national policies.^11^ However, as with survey methods, data collection is burdensome for participants and not without reporting biases.^12^ Furthermore, the cost of access is potentially prohibitive for many researchers. In acknowledgement of these limitations, Bandy et al^11^ called for a review of electronically captured sales data gained directly from supermarket retailers as an alternative objective source of food-purchase data. In this review, we aimed to address this gap and to provide an update to the previous review by Tin et al.^10^

For this review, we synthesized information from existing studies to understand the utility of electronically captured supermarket purchase records in dietary research and offer a clearer understanding of benefits of these data as well as the methodological challenges faced. This understanding will facilitate methodological innovation in dietary assessment, in turn contributing to a better understanding of dietary behaviors worldwide. Thus, we used a narrative approach to address the following questions in this review:

- What types of studies use supermarket electronic-transaction records to assess diet-related behaviors in adults?
- Are supermarket transaction data a valid dietary assessment measure?
- What sources of nutrient data did the studies use?
- What nutritional outcomes did the studies report?

## METHODS

This review is reported in line with guidance of the Preferred Reporting Items for Systematic Reviews and Meta-analyses (PRISMA) (see Table S1 in the Supporting Information online). The protocol for this review was published in advance in the PROSPERO database (registration no. CRD42018103470).^13^

### Search strategy

Four electronic journal databases—Medline, Embase, PsycINFO, and Global Health—were searched for papers published in the English language using Medical Subject Headings and keywords relating to diet or nutritional assessment and purchase data (eg, diet, nutrition assessment, grocery store, purchase, loyalty card). An example search strategy can be found in Table S2 in the Supporting Information online. Citations were imported into EndNote reference manager and titles and abstracts were independently screened by 2 reviewers (V.L.J. and F.P.) against inclusion criteria. Full texts were requested for all eligible titles and independently screened by the same 2 reviewers. A third reviewer (M.A.M.) was available throughout the screening process to resolve any disagreements. Reference lists of identified papers and hand searching were used to identify additional papers for inclusion.

### Study selection and data extraction

Studies of any design using electronically captured supermarket sales data to assess dietary outcomes (any measure) were included in this review. Studies using paper cash-register receipts or purchase data from market-research panels were excluded. Studies measuring non-nutritional aspects of diet (eg, organic, fair trade) also were excluded. Purchase data had to have been captured at the individual or household level in the general, healthy, free-living population and studies exclusively capturing purchases made by children < 18 years were excluded. Dietary outcomes for inclusion were quantity of sales at a product or category level (expressed as expenditure or volume), purchased macro- and micronutrients, and dietary patterns. The full eligibility criteria are described in Table 1.

**Table 1:** PICOS criteria for inclusion and exclusion of studies

	Inclusion criteria
Participants	Not purchases made exclusively by children <18 years old, although children may be part of the household Individuals or households Healthy (disease status unknown) Free living
Interventions	Electronically captured supermarket purchase records Purchases made at the individual or household level Not purchases made by organizations or at a national level (eg, food balance sheets)
Comparisons	Not applicable
Outcomes	Volume- or value-based food and/or beverage purchases Purchased macro- and micronutrient quantities Nutritional quality of purchased products (eg, nutrient profile) Dietary pattern derived from purchased products Electronically captured purchase records derived from supermarkets Not paper-based cash-register receipts Not self-reported purchases Not purchase records collected by market-research panels Not purchases made in laboratory-based experimental studies Not non-nutritional outcomes (eg, fair trade, organic, food safety)
Study design	Randomized controlled trial Cohort Cross-sectional Quasi-experimental Not reviews

The 2 reviewers (V.L.J. and F.P.) piloted a data extraction form, which was adapted from the Cochrane Public Health Group Data Extraction and Assessment Template,^14^ for 2 papers. This was accepted and is provided in Table S3 in the Supporting Information online. The data extraction form incorporates 2 of the key elements identified in the BEE COAST framework for reporting big data in an obesity research context^15^: description of the original data purpose and aggregation level. Data extraction was carried out by the lead reviewer (V.L.J).

### Quality assessment

The National Institutes of Health Quality Assessment Tool for Observational Cohort and Cross-Sectional Studies^16^ was used for risk-of-bias assessment. Studies were assessed by the lead reviewer (V.L.J.) against questions in 13 domain areas, which were answered “yes,” “no,” “not applicable,” or “not reported/could not determine.” The tool does not use a points system to generate an overall quality score. Instead, the answers to each of these domains contributed to an overall judgement—“good,” “fair,” or “poor”—made on the quality of study design and reporting. Studies rated “good” had a maximum of 3 domains that were not answered “yes.” “Validity of outcomes” and “adjustment for confounders” were considered the most important domains determining classification of poor study quality.

### Data synthesis

Because of the variability in study outcomes and methodologies, it was not possible to quantitatively synthesize the study data. Instead, a systematic narrative synthesis approach was used to explore findings and methods thematically, in line with the proposed research questions. The report *Guidance on Narrative Synthesis in Systematic Reviews* by the Economic and Social Research Council Methods Programme was followed.^17^

## RESULTS

### Search results

**Figure 1:**
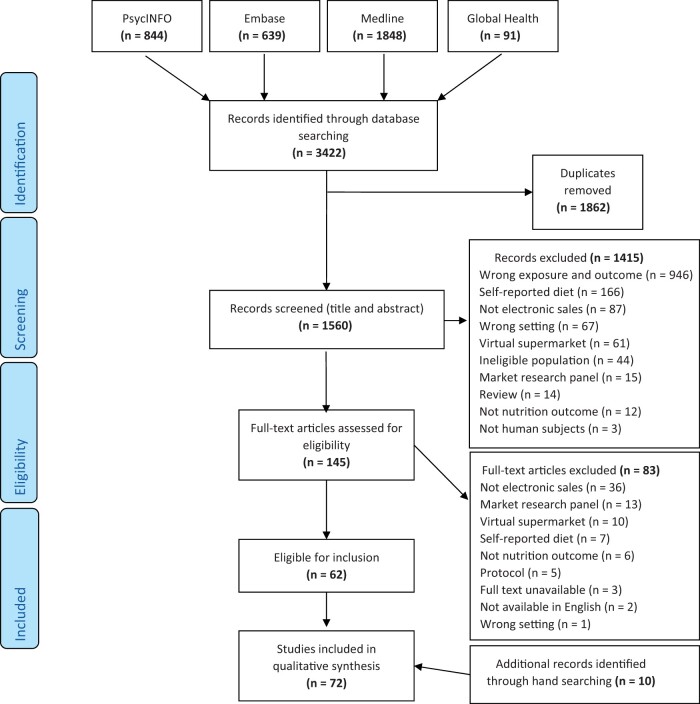
Study selection PRISMA flow chart for a review of the use of electronic sales records in population dietary surveillance (June 2020).

As the PRISMA flow chart in Figure 1 shows, the literature search returned a total of 3422 articles published between 1996 and June 2020. From these, 1862 duplicate records were removed, another 1415 articles were removed after dual screening of titles and abstracts (Figure 1). Of the remaining 145 reports that underwent full-text screening, 62 met the eligibility criteria. These were supplemented by 10 additional reports, which were identified from the reference lists of included studies, giving a total of 72 articles. A detailed summary of articles included in this review can be found in Table S4 in the Supporting Information online.

### Study characteristics

Routinely collected electronic sales data were used in 53 unique studies (*n* = 72 articles) to monitor dietary outcomes across 14 high-income countries between 1996 and 2019 (Table 2). After initial interest in the late 1990s, publication of studies using supermarket electronic purchases declined, but the number has been increasing recently. This observation reflects both the increasing availability of data and interest in its utility for dietary research.

**Table 2:** Summary of included articles’ characteristics

Characteristic	No. of papers (%)
Country	
United States	33 (46)
Australia	8 (11)
New Zealand	6 (8)
Denmark	4 (6)
Finland	4 (6)
South Africa	4 (6)
United Kingdom	3 (4)
France	2 (3)
Italy	2 (3)
Netherlands	2 (3)
Barbados	1 (1)
Belgium	1 (1)
Canada	1 (1)
Switzerland	1 (1)
Year of publication, range	
1996–2000	11 (15)
2001–2004	1 (1)
2005–2008	2 (3)
2009–2012	9 (13)
2013–2016	24 (33)
2017–2020	25 (35)
Study design	
Policy evaluation	12 (17)
In-store choice architecture	16 (22)
Financial intervention	17 (24)
Feasibility	3 (4)
Dietary surveillance	16 (22)
Comparison with intake	2 (3)
Community intervention	6 (8)
Data aggregation level	
Country/area	2 (3)
Store	25 (35)
Customer	42 (58)
Transaction	3 (4)
Socioeconomic status	
High	6 (8)
Mixed	8 (11)
Low	21 (29)
Not reported	37 (51)
Nutrient data source	
National FCDB	4 (6)
Commercial FCDB	1 (1)
Retailer (back of pack)	4 (6)
Combined	10 (14)
None	53 (74)
Duration of transaction data (months)	
0–12	46 (64)
13–24	15 (21)
25–36	6 (8)
≥ 37	5 (7)

*Abbreviation:* FCDB, food composition database.

### Risk of bias

Of the 72 articles included in this review, the majority (*n* = 42; 58%) were assessed as being of fair quality (see Table S5 in the Supporting Information online); this reflects the observational nature of many study designs. Ten articles (14%) in this review received a quality rating of good, and the remaining 20 articles (28%) were rated poor in terms of quality of study design and reporting. Dominant risks of bias across studies were poorly defined study populations, lack of justification of sample size, and the reporting of participation and follow-up rates.

### Study aims

The majority of papers used transaction data to evaluate the success of dietary interventions (*n* = 40; 56%), including: financial incentives or penalties (*n* = 18; 25%), community behavioral-change interventions (*n* = 7; 9%), or environmental nudges ,such as changes to the in-store architecture (*n* = 15; 21%) (Table 2). Twelve papers (17%) evaluated national or regional policies and 16 (22%) used observational designs for dietary surveillance (Table 2). Just 2 studies (3%) directly compared electronic transaction data with measures of dietary intake, and 3 investigated the methodological feasibility of using supermarket sales for dietary research by exploring methods for linkage with nutritional data sources (Table 2).

Intervention studies were typically short term. Consequently, the majority of studies used no more than 12 months of transaction data (Table 2). A few studies collected transaction data over several years, with a maximum duration of 8 years; these were typically policy evaluations or longitudinal dietary surveillance.

#### Evaluating intervention effectiveness.

Transaction data provided evidence for success of in-store choice-architecture interventions,^18–22^ financial interventions,^23–28^ and community interventions.^29–33^ By capturing all food purchases, transaction data revealed variation of intervention effectiveness by food category,^34^ with staple foods more resistant to change.^33^^,^^35^ Moreover, mode of intervention delivery is likely to influence effectiveness. For example, although online shopping shows promise for customization of the shopping experience, through nudge-style interventions based on previous purchases,^36^ low-income customers are less likely to shop online.^37^ Thus, online interventions could widen societal inequalities. At the individual level, intervention effectiveness may be greater than purchase estimates suggest, because the size of individual dietary changes may be attenuated by household-level purchases.^29^

#### Dietary surveillance.

Electronic point-of-sale systems generate high-volume transaction data with a fine temporal granularity. Continuous transaction data revealed how dietary patterns change over time, including monthly trends in relation to payment in low-income groups,^38^ as well as seasonally^38–40^ and longitudinally.^41^ Thus, transaction records were used retrospectively for natural experiments in policy evaluations and provided commercial insights, including market trends^42^ and price elasticities.^34^^,^^43^^,^^44^ However, the degree of insight depends on the level of data aggregation, both geographically and at the product level.

Two studies (3%) aggregated supermarket purchase data to the country level for observation of national market trends^42^ and policy evaluation.^45^ In 7 articles (10%), purchases were aggregated to the area level (city or region) to understand the effectiveness of policies,^46^ community interventions^29–31^^,^^33^ and surveillance of regional dietary variations.^44^^,^^47^ No studies explored diet at the neighborhood level, and none used geographic mapping techniques. In 25 reports (35%), authors used store-level purchases to evaluate community interventions or policies that used cluster randomization^24^^,^^29–31^^,^^48^ and quasi-experimental designs.^45^^,^^46^^,^^49–54^

In 3 studies (4%), researchers disaggregated purchases to the transaction level. This increased data volume permitting novel data-driven approaches to hypothesis generation, even without linkage to individual customers. For example, unsupervised machine learning (k-means clustering) revealed differences in dietary quality by type of alcohol purchased.^55–57^ This suggests that, in a dietary patterns context, alcohol type may be an important health consideration, perhaps as a marker for socioeconomic status, in addition to total alcohol units.

In total, 42 articles (58%), authors reported used loyalty card records to link transactions at the customer level via a unique customer identifier. Cohorts of loyalty card customers can be tracked over time, increasing confidence in observed temporal patterns and intervention effectiveness. Customer cohorts enable understanding of behavioral mechanisms and reveal within-population dietary differences and intervention responsiveness. For example, the link among socioeconomic factors, intervention effectiveness,^39^ and dietary quality^58^ suggests that restricting price promotions for unhealthy products may be more powerful for obesity prevention than discounting healthy products.^58^

In general, customer demographics were poorly described. More than half of articles did not report the socioeconomic status of participants (Table 2). Of those 42 in which loyalty card data were used, 16 studies (38%) did not report any demographic information for the customer sample, hindering assessment of generalizability. Demographic information was most commonly obtained from baseline surveys (*n* = 23; 32%), which enabled researchers to capture sensitive information, such as body mass index,^59^ education level,^23^^,^^60^ and income,^23^^,^^25^ that would not be held by the retailer. Researchers obtained demographic information from the retailer’s records for 2 studies (3%).^57^^,^^61^ Retailer-captured demographic records were limited to age, sex, and residential postcode.^57^^,^^61^ In another, researchers attempted to use supermarket-collected customer demographic information,^39^ but they was unable to do so because of poor completion of the loyalty card sign-up form. Also, customers forgetting to use their loyalty cards,^62^ and self-selection^63^ were identified as problematic for the coverage and generalizability of loyalty-card customer samples.

In the absence of customer demographic information, area-level proxies, based on store location., were used in 13 studies. For example, area geodemographics (ie, geographic segmentation based on the characteristics of people residing there)^44^ or census-tract characteristics^46^^,^^64^^,^^65^ were used to characterize the customer-base.

Other socioeconomic proxies included store type (regular or discount)^50^^,^^66^ and payment method, such that payments made with an electronic benefits-transfer card identified low-income customers in receipt of US state benefits.^36^^,^^37^^,^^65^^,^^67^ Four studies (6%) used geocoded store locations to reveal spatial and demographic variation in dietary behaviors^47^ and responses to policy interventions.^49–51^ No studies explored spatial variations in diet based on customer residential address.

#### Dietary assessment

##### Representativeness of total household food purchasing

Four studies (6%) used additional self-reported household purchase data. The findings suggested that among loyal customers, supermarkets may account for between 63% and 67% of total household food expenditure.^62^^,^^68^^,^^69^ However, shopping habits even among the most loyal customers are highly variable, resulting in wide confidence intervals for these estimates.^62^^,^^69^ In addition, data missing as a result of technical issues with electronic data capture^35^^,^^54^ and customers forgetting to use loyalty cards during shopping further reduced total purchase coverage by as much as 15%.^62^

One study (1%) compared purchase records with national expenditure surveys.^70^ The researchers reported that purchase estimates of proportional spending on staple foods fell within 2% of national expenditure surveys.^70^ However, agreement was poorer for discretionary products like sweet foods and beverages,^70^ even after excluding categories from the Household Expenditure Survey that were not covered by supermarket purchases (eg, takeaway and restaurant meals).^70^ No studies quantified the statistical agreement between household-food purchase estimates from supermarket transaction records and self-reported expenditure.

##### Representativeness of individual food consumption

Additional data on self-reported individual dietary intake were collected in 17 studies (24%). None of the studies included in the present review attempted to extrapolate absolute dietary estimates from household purchases to the individual level. Instead, dietary estimates from purchase records were represented proportionally, such as percentage contribution to total energy or expenditure.^68^^,^^70^^,^^71^ Other studies presented outcomes in terms of binary dietary behavior indicators (ie, customer-purchased the food item of interest, or did not),^47^ or diet-quality indices.^72^^,^^73^

One study (1%) directly compared household purchase estimates with individual self-reported consumption, using Spearman correlation coefficients and paired *t* tests.^71^ However, statistical agreement was not formally assessed.^71^ Another study compared nutrient availability in supermarket purchases with national dietary consumption surveys.^70^ Overall, they reported good comparability between adjusted dietary estimates from purchase records and self-reported intake.^70^^,^^71^ Yet there is evidence for variability in agreement by food type^47^^,^^70^^,^^74^ and by nutrient.^71^ Agreement was highest for energy from saturated fat and total fat. For protein, sugar, and sodium, purchase records under-reported, compared with repeated 24-hour recalls,^71^ suggesting that key food sources of these nutrients are more likely to be purchased elsewhere. In contrast with other macronutrients, estimates from purchase records were higher than self-report estimates.^71^

Comparison with national dietary-intake surveys also revealed differences in agreement within the population, with a poorer association observed for children’s diets.^70^ Having children in the house is likely to affect the types of food chosen. A positive relationship was observed between purchases of fresh produce and the number and age range of children, independent of household size.^75^ Household composition, therefore, is likely to be an important influencer of food purchasing and how products are distributed among the household, but this cannot be gained from secondary purchase records.

### Sources of nutrient data

Of the 72 included papers, 53 (74%) did not link transactions to any source of nutrient information (Table 2). Four papers (6%) used National Food Composition Databases (FCDBs) only, 3 used “Back of Pack” (BOP) product-label information, 1 used information in the product description, and 1 used a commercial FCDB. The most common approach was to combine multiple data sources (*n* = 10 papers; 14%) (Table 2), creating a custom FCDB with which purchased food and beverage products could be matched.

The source of nutrient information influences the degree of error incorporated into dietary estimates at the nutrient level. National FCDBs are used to code dietary survey responses, because they contain detailed nutrient information for commonly consumed generic foods. Yet, matching to transaction records results in reduced dimensionality from several thousand retail products to just a couple of thousand generic foods and a loss of product-specific detail.^39^^,^^76^ Furthermore, FCDBs are restricted to the most commonly consumed foods and, therefore, may poorly represent ethnic foods.^77^ This introduces greater error into nutrient-level estimates for some population subgroups. Despite these limitations, national FCDBs are readily available and enable comparison with national dietary surveys^72^ and adjustment for edible portion and specific gravity,^24^^,^^77^ improving the representation of products as eaten rather than as sold.

However, matching transaction data to FCDBs is challenging. Because of the large number and high turnover of retail products, there have been attempts to develop automated, scalable and repeatable FCDB-matching approaches. Although near-perfect matches for standard food groups may be possible, in the absence of commonly used product identifiers, there are barriers to mapping to detailed nutrient content.^24^^,^^76^ At the food-item level, string- and fuzzy-matching algorithms may be hindered by retailer abbreviations.^72^^,^^76^^,^^77^ This may be overcome if a full product description can be identified from the unique product code by web scraping.^72^ Nevertheless, in some circumstances, retailers’ short product descriptions can prove advantageous in minimizing noise from excess information that reduces match accuracy.^77^

Alternatively, nutrient data may be mapped at the category or subcategory level^76^^,^^77^ However, doing this is prone to mismatching errors resulting from different categorization approaches.^73^^,^^76^ FCDB categories are nutritionally led, whereas retailer categories are based on product placement in store and, consequently, are nutritionally heterogeneous.^73^^,^^76^ For example, a retailer “soft drinks” category, including both full-sugar and diet beverages, resulted in a mismatch of approximately 30%.^76^

Where BOP nutrient information is available from the retailer, automated linkage to the transaction record may be achieved via the unique product code.^32^^,^^68^^,^^70–72^^,^^78^ This improves product-specific nutrient accuracy and coverage of the product portfolio, which, in turn, enables between-brand comparison and reflects changes in formulation over time.^43^^,^^56^ However, the ever-evolving retail offer makes unique product codes an unstable identifier.^76^ Furthermore, lack of publicly available, digitized unique product code–level FCDBs was highlighted as a major barrier to linkage between transactions and their nutrient values.^72^^,^^76^ Although commercial data sets are available,^32^ cost- and data-sharing agreements restrict their use^72^ and their availability cannot be relied upon. Since their publication, 2 of the third-party data sources used by studies in this review are no longer available for use.^70^^,^^78^ For these reasons, a combination of nutrient data sources was typically used by researchers, generating their own FCDB.^24^^,^^39^^,^^46^^,^^65^^,^^70^^,^^72^^,^^76–79^

### Outcomes

Nutrient-level analyses^32^^,^^68^^,^^70^^,^^71^^,^^78^ focused on energy and key BOP macronutrients. With the exception of sodium (*n* = 3),^24^^,^^71^^,^^78^ no studies conducted micronutrient-level analysis. Nutrient analyses were presented in absolute terms at the household level,^78^ or more commonly were energy-adjusted, meaning that nutrient-specific dietary adequacy could not be assessed.

Because of challenges of data availability and linkage with nutrient data, most studies conducted analysis at the food category or subcategory level.^19^^,^^55^^,^^73^ As Brinkerhoff et al^76^ described, supermarket-derived categories may not be wholly meaningful from a nutritional perspective. Category-level purchases were measured in terms of relative or absolute unit sales,^20^^,^^32^^,^^35^ expenditure,^19^^,^^54^^,^^67^ or weight, volume, or portions.^23^^,^^46^^,^^49^^,^^50^ Single food products^20^ or broader categories (commonly, fruit and vegetables^24^^,^^41^^,^^68^ and soft drinks^23^^,^^45^^,^^54^) were used as outcomes for intervention and policy evaluations. Because food-purchase decisions are not independent of each other, this approach may miss unintended negative consequences such as substitution effects within other categories.^63^^,^^64^ For this reason, Taylor et al^73^ advocated a broader dietary-pattern view to examine dietary quality.

The study of dietary patterns involves classifying customers into groups on the basis of their purchasing habits. Groups may be defined a priori on the basis of the purchase of a product of interest, in a deterministic approach. For example, Johansen et al^56^ used a dichotomous approach based on whether different alcoholic beverages were purchased or not, to classify customers as wine buyers, beer buyers, purchasers of a mixture of alcoholic beverages, or non-alcohol purchasers. Or groups may be defined on the basis of the dietary quality of products purchased. Products may be classified on evidence of diet-disease relationships,^55^ professional opinion,^35^ or using custom or established nutrient profile models.^28^^,^^32^^,^^39^^,^^58^^,^^72^^,^^73^ However, in many cases, classification criteria were not transparently described for reproducibility. Established nutrient profile models use predefined criteria, making them stable metrics for dietary surveillance (eg, in assessing compliance with dietary guidelines). Classification of products shows that shoppers prioritize purchases of “unhealthy” food products over “healthy” foods.^55^^,^^73^ The majority of expenditure was on discretionary foods (34.8%), followed by meat and meat alternatives (17.0%), with the least spent on vegetables and dairy products.^73^ Vandenbroele et al^20^ advocated that retailers shift from product-focused thinking to a whole-basket approach. Focusing on overall-purchase dietary quality will enable retailers to implement choice-architecture strategies that maximize health as well as profits.

Alternatively, dietary patterns may be explored nondeterministically through unsupervised machine-learning algorithms. Hansel et al^55^ used K-means clustering to classify customers according to their alcohol-purchasing habits. Not only does this approach account for frequency and quantity, it revealed greater dietary nuance between alcohol-purchasing groups, such as the specific dietary habits of purchasers of aniseed-based beverages and Bordeaux wines. Researchers observed a relationship between purchases of beer and the less healthy, traditional-type diet and between purchases of wine and the healthier Mediterranean-type diet,^55^^,^^56^ highlighting the utility of dietary patterns for describing dietary quality, although they could quantify it.

## DISCUSSION

The 2017 Review of Nutrition and Human Health Research^80^ describes a field in crisis. The review highlights the limitations of self-reported dietary intake methods, which, it is argued, contribute to a perceived lack of rigor in nutrition research.^80^ The inability to accurately measure diet has damaged confidence in nutrition research findings. Consequently, there has been little progress toward improvements in population diet, despite substantial efforts from interventions and policy.^81^

There is no gold standard method of dietary assessment that can answer all diet-related questions. The breadth of questions posed by the field of nutrition research, therefore, requires a suite of innovative methods to supplement existing approaches. This necessitates the harnessing of technology and secondary data sources, where they are available. Just as biomarkers complement surveys with an objective measure of nutrients within the body, supermarket transaction records provide a complementary objective measure of food purchases.

In this review, we found that supermarket electronic purchase records can be useful for longitudinal dietary surveillance^74^ in high- and middle-income populations where supermarket shopping is prevalent and represents the majority of household expenditure.^62^^,^^68^^,^^69^ Transaction data have a number of strengths. Large data volumes enable data-driven exploration of dietary patterns^55–57^^,^^82^ to better understand food-purchase behaviors and identify intervention target groups. Furthermore, continuous data collection permits observation and control for day to day,^83^ week by week,^38^ and seasonal variation in dietary choices,^59^ which cannot be revealed in such detail by cross-sectional dietary surveys.

Large customer samples and passive data collection may improve representation of hard-to-reach groups. This was demonstrated by good diffusion across income groups within a single-retailer sample in the United Kingdom^84^ despite being unrepresentative of the general population overall.^85^ Similarity to regional dietary estimates from survey data^47^ highlights the utility of electronic transaction records for within-country ecological studies of diet. To date, much research on spatial variation in diet has focused on the food environment,^86^ such as accessibility to supermarkets^87^ or fast-food outlets,^88^ rather than actual behaviors. Although spatial patterns may be observed in dietary survey data,^89^ large sample sizes are required to reduce the risk of ecological fallacy; as such, aggregation areas tend to be large. Using store location and, where available, customer area of residence (as geocoded reference points), the scale of supermarket electronic purchase data enables small-area spatial analysis,^84^^,^^90^ provided this is permitted by data usage agreements, given the proprietary nature of retailer data, and that appropriate information management systems are in place.

Limitations of supermarket electronic transaction data are partial coverage of total food purchased or otherwise obtained, unknown distribution of food within households, and inability to account for food wasted or food consumed by visitors.^91^ As such, household-level purchase data do not directly measure individual dietary intake.^71^ Findings of studies in this review suggest, at best, moderate agreement between household purchase and individual intake estimates.^70^^,^^71^ Given these limitations, there is a need for validation against existing methods to better understand the utility of supermarket transaction records for monitoring dietary behaviors. Triangulation with other dietary assessment methods may reveal additional insights and enable generation of adjustment factors for improved consumption estimates.

Statistical agreement^92^ between electronic purchase records and self-reported methods was not formally assessed by studies in this review. However, observed correlations^70^^,^^71^ support this review’s ability to capture the majority of the diet. This adds weight to earlier work that found good agreement between estimates of fat and energy from paper-based cash-register receipts and self-reported 4-day food diaries.^4^ Just how much purchase data are required to represent habitual diet warrants further exploration, but evidence from this review suggests that approximately 7 days of transaction records may be enough to represent usual diet,^83^ at least for perishable, high-turnover products.

Because no studies in this review attempted to adjust household purchases to the individual level, it is unclear how well household purchases represent the diet of individuals within a household. Modelling individual diet from household purchases would require several assumptions and necessitate further study.^91^ To do so, additional survey information^4^ about household composition and within-household food distribution would be needed to adjust for person-specific measurement error.^91^ Alternatively, modelling techniques, such as microsimulation^93^ and other mathematical approaches, may offer a means to estimate diet at the individual level. Transaction data can contribute to refinement of modelling parameters (eg, understanding the impact of age of children in the household on fruit and vegetable purchase quantities).^75^

There has been increasing recognition of the importance of engaging with the food industry to translate research insights into action. Effective research-industry partnerships, therefore, are vital, as explained by the guidance framework proposed by Birkin et al.^94^ Although the studies in this review did not explicitly discuss the challenges associated with partnership building, it is a key consideration for researchers wishing to harness the potential of supermarket transaction records. That said, challenges relating to the way data are shared can contribute to the sources of bias we observed through this review: a lack of information about the study participants, lack of transparency in recruitment, and inability to control for customer demographic characteristics, which might act as confounders for dietary behaviors.

New approaches to transparency and customer consent are warranted to enable greater utility of customer-level data. Efforts are needed to overcome issues of poor data quality,^39^^,^^62^ restricted information,^57^^,^^61^ and assessment of customer-sample bias. Innovations such as the Danish Data for Good Foundation’s platform, which enables bespoke customer informed consent and triangulation of public- and private-sector data, could offer a potential solution.^95^

Another obstacle for the future of the method is the lack of centralized and up-to-date, product-level FCDBs, which may be linked to automatically.^91^ Studies in this review reported the need to create new bespoke FCDBs to facilitate linkage with nutrition information. This requires a substantial amount of up-front resources, which limit time to generate interesting research insights. Although commercial FCBDs exist, cost- and data-sharing agreements can be a barrier. Furthermore, their coverage is typically limited only to those nutrients required to be reported by local BOP labelling regulations, which contributes to a lack of utility for micronutrient monitoring and differences in nutrient coverage between countries. In contrast, national food tables are freely available and cover a wider range of nutrients, but for fewer and more generic foods.

Solutions could include the linkage of product data to close-matching generic foods in national FCDBs, which contain detailed micronutrient compositions, as performed by the dietary assessment app *myfood24*.^96^ Yet, ensuring FCDBs stay up to date remains a challenge. Innovations such as foodDB^97^ harness web scraping to provide regularly updated BOP nutrition-composition information for products on the market. It is also possible that 3-dimensional barcode advances, which permit greater data capture, may further improve product-level FCDBs through the inclusion of micronutrient information and supply-chain data, such as origin and sustainability metrics. Viable country-specific FCDB solutions, therefore, are vital to enable nutrient- and brand-level research insights from supermarket transaction data, which this review found to be lacking. In addition, there is a role for bodies such as the Food and Agriculture Organization’s International Network of Food Data Systems^98^ to develop global standards for the reporting and exchange of product nutrient data to promote consistency and facilitate across-country comparison.

### Future research priorities

This review highlights 5 priority areas for research into the use of supermarket sales data for population dietary surveillance: 1) validation against established self-report methods and nutritional biomarkers; 2) extrapolation of household purchases to the individual level; 3) triangulation with other data sources; 4) exploration of spatial dietary patterns; and 5) development of suitable nutrient data sets for linkage.

## CONCLUSION

Our findings from this review suggest electronic purchase records have broad applicability for dietary surveillance, policy evaluation, and intervention research studies in high- and middle-income countries. The scale, temporality, and geocoded nature of electronic purchase records are notable advantages. However, there is a need for additional methodological assessment of utility; validation against self-reported dietary intake measures and nutritional biomarkers; required data volumes; extrapolation to the individual level; exploration of spatial dietary patterns; and assessment of generalizability. The potential for automated dietary coding is currently hindered by the availability of regularly updated, open product data. Web-scraping methods may address this need. However, this lack limits coverage to key BOP nutrients, which exclude micronutrients (with the exception of sodium). Product data alone account only for dietary availability; linkage with sales data is crucial for behavioral research.

## Supplementary Material

nuab089_Supplementary_Data

## Supporting Information

The following Supporting Information is available through the online version of this article at the publisher’s website.

*Table S1* **PRISMA checklist**

*Table S2* **Example search strategy (MEDLINE; OVID interface, 1996 onward)**

*Table S3* **Data extraction form**

*Table S4* **Summary of included studies**

*Table S5* **National Institutes of Health Quality Assessment Tool for Observational Cohort and Cross-Sectional Studies risk-of-bias assessment for included papers**
