# Supplementary material for: A systematic review of supermarket automated electronic sales data for population dietary surveillance
Source: Nutr Rev. 2022 May 5;80(6):1711–22. doi: 10.1093/nutrit/nuab089 (PMC9086796; doi:10.1093/nutrit/nuab089)
Supplement: nuab089_Supplementary_Data [file nuab089_supplementary_data.docx]

Table S1. PRISMA Checklist

| **Section/topic** | **#** | **Checklist item** | **Reported on page #** |
| --- | --- | --- | --- |
| **TITLE** | | |  |
| Title | 1 | Identify the report as a systematic review, meta-analysis, or both. | 1 |
| **ABSTRACT** | | |  |
| Structured summary | 2 | Provide a structured summary including, as applicable: background; objectives; data sources; study eligibility criteria, participants, and interventions; study appraisal and synthesis methods; results; limitations; conclusions and implications of key findings; systematic review registration number. | 1 |
| **INTRODUCTION** | | |  |
| Rationale | 3 | Describe the rationale for the review in the context of what is already known. | 2 - 4 |
| Objectives | 4 | Provide an explicit statement of questions being addressed with reference to participants, interventions, comparisons, outcomes, and study design (PICOS). | 4 |
| **METHODS** | | |  |
| Protocol and registration | 5 | Indicate if a review protocol exists, if and where it can be accessed (e.g., Web address), and, if available, provide registration information including registration number. | 4 |
| Eligibility criteria | 6 | Specify study characteristics (e.g., PICOS, length of follow-up) and report characteristics (e.g., years considered, language, publication status) used as criteria for eligibility, giving rationale. | 4, (Appendix 3) |
| Information sources | 7 | Describe all information sources (e.g., databases with dates of coverage, contact with study authors to identify additional studies) in the search and date last searched. | 4 |
| Search | 8 | Present full electronic search strategy for at least one database, including any limits used, such that it could be repeated. | Appendix 2 |
| Study selection | 9 | State the process for selecting studies (i.e., screening, eligibility, included in systematic review, and, if applicable, included in the meta-analysis). | 5 |
| Data collection process | 10 | Describe method of data extraction from reports (e.g., piloted forms, independently, in duplicate) and any processes for obtaining and confirming data from investigators. | 5 |
| Data items | 11 | List and define all variables for which data were sought (e.g., PICOS, funding sources) and any assumptions and simplifications made. | Appendix 4 |
| Risk of bias in individual studies | 12 | Describe methods used for assessing risk of bias of individual studies (including specification of whether this was done at the study or outcome level), and how this information is to be used in any data synthesis. | 5 – 6, Appendix 6 |
| Summary measures | 13 | State the principal summary measures (e.g., risk ratio, difference in means). | N/A (narrative synthesis) |
| Synthesis of results | 14 | Describe the methods of handling data and combining results of studies, if done, including measures of consistency (e.g., I^2^) for each meta-analysis. | N/A |

| Risk of bias across studies | 15 | Specify any assessment of risk of bias that may affect the cumulative evidence (e.g., publication bias, selective reporting within studies). | 7 |
| --- | --- | --- | --- |
| Additional analyses | 16 | Describe methods of additional analyses (e.g., sensitivity or subgroup analyses, meta-regression), if done, indicating which were pre-specified. | N/A |
| **RESULTS** | | |  |
| Study selection | 17 | Give numbers of studies screened, assessed for eligibility, and included in the review, with reasons for exclusions at each stage, ideally with a flow diagram. | 6 (Figure 1) |
| Study characteristics | 18 | For each study, present characteristics for which data were extracted (e.g., study size, PICOS, follow-up period) and provide the citations. | Appendix 5 |
| Risk of bias within studies | 19 | Present data on risk of bias of each study and, if available, any outcome level assessment (see item 12). | Appendix 6 |
| Results of individual studies | 20 | For all outcomes considered (benefits or harms), present, for each study: (a) simple summary data for each intervention group (b) effect estimates and confidence intervals, ideally with a forest plot. | 7-15 |
| Synthesis of results | 21 | Present the main results of the review. If meta-analyses are done, include for each, confidence intervals and measures of consistency. | Appendix 5 |
| Risk of bias across studies | 22 | Present results of any assessment of risk of bias across studies (see Item 15). | 7 |
| Additional analysis | 23 | Give results of additional analyses, if done (e.g., sensitivity or subgroup analyses, meta-regression [see Item 16]). | N/A |
| **DISCUSSION** | | |  |
| Summary of evidence | 24 | Summarize the main findings including the strength of evidence for each main outcome; consider their relevance to key groups (e.g., healthcare providers, users, and policy makers). | 16 – 23 |
| Limitations | 25 | Discuss limitations at study and outcome level (e.g., risk of bias), and at review-level (e.g., incomplete retrieval of identified research, reporting bias). | 16 – 23 |
| Conclusions | 26 | Provide a general interpretation of the results in the context of other evidence, and implications for future research. | 23 |
| **FUNDING** | | |  |
| Funding | 27 | Describe sources of funding for the systematic review and other support (e.g., supply of data); role of funders for the systematic review. | 24 |

Table S2. Example search strategy (MEDLINE; OVID interface, 1996 onwards)

| **#** | **Search Term** |
| --- | --- |
| 1 | diet$.mp or DIET/ or “DIET, FOOD AND NUTRITION”/ |
| 2 | diet records.mp or Diet Records/ |
| 3 | energy intake.mp or Energy Intake/ |
| 4 | food.mp |
| 5 | diet quality.mp |
| 6 | Nutrition Assessment/ or dietary assessment.mp |
| 7 | food supply.mp or Food Supply/ |
| 8 | (food adj purchas$).mp |
| 9 | (diet surveys or nutrition surveys).mp |
| 10 | nutrition monitoring.mp |
| 11 | ((food or diet$) adj habit$).mp |
| 12 | or/1-11 |
| 13 | Commerce/ or supermarket$.mp |
| 14 | grocery store$.mp |
| 15 | shop$.mp |
| 16 | food industry.mp or Food Industry/ |
| 17 | or/13-16 |
| 18 | sale$.mp |
| 19 | purchas$.mp |
| 20 | (scan$ adj data).mp |
| 21 | receipt$.mp |
| 22 | (loyalty adj card).mp |
| 23 | or/18-22 |
| 24 | and/12, 17, 23 |
| 25 | Limit 24 to “all adult (19 plus years)” |

Table S3. Data extraction form

| Study intention | Study aims  What was the study designed to assess?  Are the aims clearly stated? |
| --- | --- |
|  | Describe location & setting.  Might this target/exclude certain groups? |
|  | Start and end date of study |
|  | Total study duration |
| Methods | Method of participant recruitment (does this differ by setting?) |
|  | Inclusion/exclusion criteria for participation |
|  | Representativeness of sample: are participants likely to be representative of the target population? |
|  | Total number of (intervention) groups |
|  | Sample size (for each group) |
|  | Was randomisation used? If so, what unit (individuals or cluster/groups)? |
|  | Unit of analysis? Aggregation level, geographic unit. (Where applicable, was this the same as the randomisation unit?) |
|  | Describe intervention / control conditions where relevant (setting, theory, delivery, timing etc) |
|  | Statistical analysis methods used. Were these appropriate? |
|  | If secondary analysis, what was the original data purpose & context? Could this introduce bias? |
| Results | What percentage of participants agreed to participate? |
|  | Were there any significant baseline imbalances between groups? |
|  | What percentage of participants completed the study? |
|  | Describe participant characteristics (for each group) |
|  | Definition of outcome(s) including units of measurement and unit of aggregation if relevant |
|  | How were outcomes measured? |
|  | Time points measured |
|  | Results |
| Other relevant information | Potential for author conflict? Would one outcome benefit authors/data collectors? |
|  | Author's key conclusions |
|  | Comments from review authors |

Table S4. Summary of included studies

| **Study** | **Author Year** | | **Design** | **Aim** | **Setting** | **Population** | **Duration** | **Data source** | **Nutrition data source** | **Outcomes** | **Analysis** | **Key findings** |
| --- | --- | --- | --- | --- | --- | --- | --- | --- | --- | --- | --- | --- |
| **Policy evaluations** | | | | | | | | | | |  |  |
| Nutrition Labelling and Education Act (NLEA) | | Mathios (1998) | Cross-sectional | Impact of NLEA on type of cooking oil purchased | USA, New York State  20 stores | Customer number unknown  Loyalty card demographics used for sampling to ensure breadth – based on educational attainment. | 2 years, collected every 4 months (Oct 1992 – Oct 1994) | Store-level | Product nutrition labels | Market share-weight (units sold) of fat in oils (saturated, mono-unsaturated, poly-unsaturated) | Econometric model, regression analysis | Saturated fat increased all stores. Mono-unsaturated declined 17/20 stores.  Least educated increased saturated fat and most educated increased mono-unsaturated. |
|  |  | Mathios (2000) | Quasi-experimental | Impact of NLEA on sales of salad dressings |  |  |  |  |  | Market share (units sold)/ week  Fat (g/serve)  % products with voluntary nutrition label | Correlation between per serving fat and calories  Econometric model, regression | Correlation 97%  Sales of unlabelled products higher for less educated supermarkets  Greater reduction in market share for products highest in fat |
|  |  | Balasubramanian and Cole (2002) | Longitudinal | Impact of NLEA on sales of products with specific nutrition descriptors e.g. low fat | USA, several stores, major grocery chain in large city | Number & customer demographics unknown | 7 years 8 months, weekly sales | Store-level weekly scanner data | Claim in category or product description Y/N | Category share with 10 week moving average | Regression analysis | Increased sensitivity to negative nutrient claims, purchases of positive nutrient products declined or stable |
| European School Fruit Program | | Brunello et al. (2012) | Controlled before and after | Effect of EU School Fruit Program on sales of unhealthy snacks | Italy  44 stores, 2 retailers (one discount, one regular) | 15 treatment stores (within 500m of treated school) Year 1 n=100  29 control stores, Year 1 n=479, Year 2 n= 405 | 2 years (Jan 2009 – Sept 2011)  1-year pre- & 1-year post | Aggregated store-level sales data | N/A | Mean daily store sales of unhealthy sweet and salty snacks (units, kg) | Difference in differences  Regression | Treated stores 4.6% reduction in snack vs control (not significant)  Significant reduction in high income areas (-12%), regular stores (-13%) and branded products (-13%). No effect in low income areas and discount stores. |
|  |  | Brunello et al. (2014) |  |  |  |  |  |  |  |  |  |  |
| Berkeley sugar tax | | Silver et al. (2017) | Interrupted time series | Impact of Berkeley sugar sweetened beverage (SSB) tax (1 cent/ounce) on sales, price & intake | USA  26 stores Berkeley California;  3 Berkeley intervention stores, 6 control stores outside Berkeley | N = 957  Adults living in Berkeley. Affluent city with high education and low baseline SSB intake | 3 years  Pre- and post-taxation | Daily point of sale data  Store price surveys  2 repeated 24-hr dietary recall telephone surveys | Nutrition data from product website, nutrition facts panel from Mintel, USDA database | Changes in inflation-adjusted prices (cents/ounce) for taxed SSBs, sales (ounces), customer spend/ transaction, intake (g/day and kcal/day) | Difference in difference  OLS regression volume & revenue per transaction for Berkeley vs non-Berkeley stores | Taxed sales fell by 9.6%, untaxed rose by 3.5%. No change in customer spending or store revenue.  Mean intake (g) reduced by 19.8% & calories from SSBs fell by 13.3% (both non-significant) |
| Special Supplemental Nutrition Program for Women, Infants, and Children (WIC) | | Andreyeva et al. (2012) | Cross-sectional | Compare non-alcoholic beverage purchases for WIC vs SNAP benefit recipients | USA  Large supermarket chain, several New England states | 39,172 loyalty card holders  Low income young families eligible for federal food & nutrition assistance | 6 months (January – June 2011) | Loyalty card scanner data | Gladson’s Nutrition Database + internet searches | Refreshment beverage purchases/hh/month | Generalised linear regression from Poisson family with logarithmic link function | 64% matched to nutrient data  SNAP household purchased more (689 oz) than WIC (352 oz) and more SSB (58% vs 58% respectively)  SNAP paid for 72% of SSBs, ~ $1.7 – $2.1 billion/year |
|  |  | Andreyeva et al. (2013) | Natural experiment | Effect of reduced juice allowance for the Women, Infants and Children (WIC) programme | USA  >60 stores from one chain in Connecticut & Massachusetts | 2137 households  Loyalty card holders  Low income young families eligible for WIC | 20 months (January 2009 – September 2010) | Loyalty card scanner data | N/A | 100% juice purchases (floz/hh/month) by payment type (%) | Generalised linear regression from Poisson family with logarithmic link function | Total juice declined 23.5% (21.4% - 25.4%)  Reduction in 100% juice & WIC proportion. Small increase in non-WIC juice, fruit drinks, & non-carbonated. 12% (8.1% - 15%) decline in soft drinks |
|  |  | Andreyeva and Luedicke (2013) |  | Impact of including whole-grain products in WIC on purchases of bread & rice |  |  |  |  | Gladson’s Nutrition Database, internet searches, My Pyramid Equivalents Database | Bread (whole grain 100%, 51-99%, 1-50%/white) & rice (brown or white) purchases/hh/month |  | 100% whole grain bread share tripled; 8% - 24%. White bread fell; 58% - 50%. Overall bread stable. Decline in non-WIC  Brown rice share rose (0.3 Oz to 2.4 Oz), rise in white & total rice |
|  |  | Andreyeva et al. (2014) |  | Impact of reduced WIC milk & cheese allowance & disallowance of whole milk over 23 months |  |  |  |  | N/A | milk (floz) & cheese (oz) purchases/hh/month, share of whole milk, saturated fat from milk & cheese (g) |  | 13% reduction in total milk and 20% reduction in WIC-milk purchases.  Significant reduction in whole milk share and 40% reduction in WIC-eligible cheese purchases |
|  |  | Andreyeva and Luedicke (2015) |  | Impact of WIC fruit & veg vouchers on fruit & veg purchases |  |  |  |  | N/A | fruit & veg purchases/hh/month (weight, cup equivalents & expenditure) |  | Fruit & veg increased significantly (+17.5% & +28.6% respectively) P<0.001 |
| Barbados Sugar Tax | | Alvarado et al. (2019) | Interrupted Time Series | Impact of 10% added value tax on Sugar Sweetened Beverages (SSBs) | Barbados | Barbados shoppers – demographics unknown | 3 years 10 months | Country-level sales from one grocery chain | N/A | Weekly sales volume (mL) per capita SSBs and non-SSBs | Interrupted time series, linear regression | SSB sales decreased 4.3% (-4.9, -3.6%)  Non-SSB sales increased 5.2% (4.5, 5.9%) |
| **Financial interventions** | | | | | | | | | | |  |  |
| Supermarket Healthy Options Project (SHOP) | Mhurchu et al. (2007) | | Pilot RCT | Promote healthier purchases: culturally appropriate nutrition education & 12.5% price discount | New Zealand  5 Shop ‘N Go stores | 95 hhs  Age (µ) 40yrs, 72% female, 7% Maori, 2% Pacific, 91% European / other | 6 months (12 weeks baseline, 12 weeks intervention) | Self-scan transactions | N/A | Total hh food expenditure & fruit and veg purchases | Participant descriptive statistics  Analysis of shopping diaries | Poor enrolment by minority ethnic groups.  Supermarkets = 66% total expenditure (51% captured by Shop ‘N Go system, 33% at other retailers) |
|  | Mhurchu et al. (2010) | | RCT | Effect of 12.5% price discount & tailored nutrition education on food & nutrient purchases | New Zealand  8 Shop ‘N Go stores | 1,104 households, Age (µ) 44yrs, 86% female, 22% Maori, 9% Pacific, 68% European/ other, 52% low income, 51% low qualification | 12 months + 12 weeks baseline  (24 weeks intervention, 24 weeks follow up) |  | Supermarket Food & Nutrition Database (SFND); Manufactured Food Database, brand websites, back of pack & NZ food tables | % hh food energy from saturated fat (other macro-nutrients secondary)  purchases of ‘healthier’ food (kg/hh/wk), | Repeated-measures mixed-model (difference from baseline) regression  Intention to Treat analysis | Increased healthy food, & fruit & veg purchases (+11% & +15% respectively) 6 months vs baseline, no difference in saturated fat or other macronutrients |
|  | Blakely et al. (2011) | | RCT |  |  |  |  |  |  |  | Sensitivity analysis by SES (ANCOVA) | Effect varied by ethnicity (non- significant); Maori -0.15kg/wk (CI -1.10, 0.8), Pacific +1.20kg/ wk (CI 0.06, 2.23), European/other +1.02kg/wk (CI 0.60, 1.43) |
| Supermarket Healthy Eating for Life (SHELf) | Ball, K. et al. (2015) | | RCT | Cost-effectiveness of tailored skill-building & price reduction to promote purchase & consumption of healthy foods & beverages among high- & low-SES women | Australia, Coles stores  2 target stores | N = 574 female loyalty card holders, Age (µ) 43.7yrs, 44.4% low SES catchment, 50.1% tertiary education, 28.6% born outside Australia | 3-month intervention, 6 months follow up, 3-month retrospective baseline data | Loyalty card transactions  FFQ & self-reported soft drink portions, Questionnaire | N/A | Purchase & consumption/ hh/wk of fruit & veg (g), sugar-sweetened & low-calorie soft drinks, water (serves, ml), self-efficacy & perceived affordability | Generalised Estimating Equations  Mediation analyses (MacKinnon method) | Increased fruit purchases at 3 months +35% (2.4 serves/wk) & veg +15% (3.1 serves/wk) vs control. Self-reported fruit consumption increased (+2.43 serves/wk). No increase in diet beverages or water.  No difference by income or education |
|  | Le et al. (2016) | | RCT |  |  |  |  |  |  | ICER (incremental cost-effectiveness ratio) A$/ additional serve | Bootstrapping with 1000 resamples. Cost-effectiveness plane. | Price Reduction: ICER = $2.3 per extra serve veg/wk , $3.0 per extra serve fruit/wk  Combined: ICER = $11.6 per increased fruit serve/wk |
| NYC supermarket discount | Geliebter et al. (2013) | | RCT | Effect of 50% price discount on purchase & intake of low-energy density fruit & veg, bottled water & diet sodas, & body weight | USA  2 stores Manhattan, New York (~1 mile apart) | N = 47 loyalty card holders, 70% female, BMI (µ) 30.2, Age (µ) 37.5, 56% Caucasian, 19% African American, 13% Hispanic  28 Intervention, 19 Control | 16 weeks  (4 weeks baseline, 8 weeks intervention, 4 weeks follow up) | Loyalty card transactions, continuous over 16-weeks  5 repeated 24-hr recalls (4 weeks apart) | N/A | Gross hh expenditure ($/wk), intake (g, kcal & servings of fruit & veg /day, 1 serve = 80g)  Body weight (kg), BMI, Body fat % | ANOVA with repeated measures (95% significance level) | Fruit & veg purchases in discount group increased 3x vs control, intake +1.5 serves. Purchases & intake significantly correlated (r = 0.62).  No difference in beverage purchase or intake |
|  | Bernales-Korins et al. (2017) | | RCT | Effect of 50% discount on purchase & intake of fruit & veg |  | N = 45 loyalty card holders |  | As above plus psychosocial measures (determinant of intake) |  | As above, plus self-efficacy, stages of change & perceived barriers | As above, plus structural equation modelling | Discount increased self-efficacy & stages of change but no change in perceived barriers. |
| SHOP@RIC (Stores Healthy Options at Remote Indigenous Communities) | Brimblecombe et al. (2017) | | RCT | Effect of 20% price discount on food & drink purchases with & without consumer education | Australia  20 remote indigenous communities with single store (2 retailers, ALPA & OBS) | Combined population ~8,515 people  10 stores discount, 10 stores discount + education | 2.5 years  19 weeks baseline, 24 weeks intervention, 24 weeks post-intervention | Sore-level weekly sales data | Food Standards Australia & New Zealand Australian Food, Supplement and Nutrient Database 2011-13 | Primary = per capita daily weight (g) fruit & veg purchased  Secondary = beverages, healthy/ unhealthy foods (g/day) | Mean difference (name of test not given) | Increased fruit & veg during (+12g/capita/ day) & after intervention (+18g/ capita/day). More effective for fruit. Additional benefits for veg with education.  Increase in total beverage, total unhealthy products, sodium & energy too |
| Outback Stores | Ferguson et al. (2017) | | Natural experiment | Evaluate price strategies; reduced grocery mark-up, fruit & veg scales, fruit & veg sold at landed cost, diet soft drink discount | Stores in 18 remote Aboriginal communities, Central & Northern Australia | 18 stores, 54 interview participants, 78% aboriginal, 89% over 35 years, 48% male | 18 months (July 2009 – December 2010) | Store-level monthly sales data | N/A | Change in grocery sales ($) ratio of total sales, fruit & veg sales, soft drink sales | Mixed effects model with random effect intercept, adjusted for correlation of monthly sales with same store  Autoregressive model, controlling for season | No impact on sales/turnover of grocery, fruit & veg or soft drinks. |
| Buywell trial | Stead et al. (2017) | | RCT | Assess impact & feasibility of targeted price promotion & healthy eating advice on targeted healthy foods | Scotland, UK  Low income areas | N = 53,363 loyalty card customers who purchase unhealthy products, 31 – 65 years  37,034 intervention, 16,333 control | 6 months  2 months baseline, 1- month intervention, 3 months follow-up | Loyalty card EPOS transactions | FSA traffic light scheme & nutrient profiling used for population sampling only | No. & % customers purchasing targeted healthy products,  Product switching | Chi-squared | Significant increase in proportion purchasing 4/5 targeted products. No significant increase for fruit & veg  8% customers switched to lower fat milk during intervention. Effects not sustained. |
| Sylacauga Aliance for Family Enhancement (SAFE) | Banerjee and Nayak (2018) | | RCT | Effectiveness of targeted education & price discount on purchases of healthy food | Alabama, USA  2 local stores | N = 100 low income families  83% female, 31% Caucasian, 65% African American, Age (µ) 39 yrs, 70% unemployed  25 Education, 25 Coupon, 25 Combined, 25 Control | 1 week | Scan data linked with store card, issued specifically for study with $40 credit | Food-A-Pedia or product nutritional label | Change in kcal/hh, sodium, added sugar, saturated fat & fibre (alcohol & non-food items excluded) | Linear regression | Education & combined significantly reduced total calories (235 & 280kcal) respectively, vs control. Coupon reduced by 97kcal relative to control (non-significant) |
| Healthy Food Program | Sturm et al. (2013) | | Case-control | Effect of 10% & 25% price reduction of healthy food on household shopping behaviours | South Africa  >400 stores (single retailer) | Members of private health insurance Healthyfood programme; No rebate: N ~ 67,794, 10% rebate: N ~ 33,558, 25% rebate: N ~ 68,133  No demographics | 3 years (2009 – 2012) | Purchases from eligible supermarkets using specific credit card, for linkage with health record & rebate  Health Risk Assessment Survey (HRA) | N/A | Ratio of healthy, fruit & veg, neutral, less-desirable, to total spend /hh/month  Intake servings fruit & veg, & whole grain, salt, sweet foods, processed meat & fast food.  Self-reported weight & height | Household fixed-effects model & case-control difference-in-differences  Sensitivity analysis; proximity to eligible stores & customer loyalty | Negligible bias by payment type or strategic shopping  10% rebate: +6% healthy, +5.7% fruit & veg, -5.6% less-desirable  25% rebate: +9.3% healthy, +8.5% fruit & veg, -7.2% less-desirable |
|  | An (2014) | |  |  |  |  |  |  |  |  | Descriptive statistics: two-sample t-test with unequal variance | Members ate more fruit & veg (+0.7 serves), 8% more likely to meet wholegrain guidelines, 2% less likely to eat high sugar, fried foods (-9%), salt (-2%), processed meat (-7%) & fast food (-8%) |
|  | Schwartz et al. (2014) | | RCT | Effect of voluntary self-control financial commitment (forfeit 25% healthy food rebate) on healthy food purchasing | South Africa  >400 stores | N = 4,073 households, members of private health insurance programme, no demographic information, 62% completed | 12 months (6 months intervention + 6 months baseline) | Transactions at FlyBuys supermarket using Discover-Health visa credit card |  | Household purchases (%) of healthy/ neutral/ unhealthy foods (units & expenditure) | Intention to treat analysis, random-effects linear regression | 36% hhs accepted pre-commitment  Pre-commitment hhs increased healthy purchases by 3.5%. No change among control or those who declined |
| Healthy Incentives Pilot (HIP) | Bartlett (2014) | | RCT | Effect of financial incentives (30% point of sale rebate) for benefit recipients, on consumption of fruits, veg & other healthy foods | USA, Hampden County, Massachusetts  130 intervention stores | 55,095 SNAP households; 7,500 intervention group (HIP), 47,595 control group (non-HIP)  Mean age 43, 73% female heads of household, ~50% Hispanic | 1 year (2011 – 2012) | Electronic Benefit Transfer Card (EBT)  Self-reported consumption & spend; telephone 24-hour recalls | N/A | Consumption (cups/day) on targeted fruit & veg (TFV)  EBT ($) on TFV  Self-reported ($) on total fruit & veg | Regression-adjusted differences between HIP and non-HIP groups | HIP consumed 0.25 cups more TFV/day (+26%), spend $6.15 more/month, & +$1.19 more EBT spend on TFV than non-HIP |
| Reducing SSB consumption in Latino community | Franckle et al. (2018) | | RCT | Effect of financial incentive and traffic light labelling scheme on reducing purchases of sugar-sweetened beverages | USA, Boston, Massachusetts  1 store | 148 households with children under 18yrs, shopping in low-income community  Intervention: 100% female, 34% over 40 years  Control: 97% female, 34% over 40 years | 2 months baseline, 5 months post-intervention | Study-specific loyalty card  Exit interview, self-reported consumption | N/A | % customers purchasing beverages labelled with red traffic light (>12g sugar/12oz serve) each month  Binary outcome ≥1 serve or none | Logistic regression | Difference in purchases of red-labelled beverages between groups (p=0.002).  Intervention group had larger reduction in purchases (-9 percentage points) and consumption (-22 percentage points) (p=0.01) |
| Healthy Double Study | Polacsek et al. (2018) | | RCT pilot | Determine if supermarket 2 for 1 on fruit and vegetables (FV) increases purchases among low-income families | USA, rural community in Portland, Maine  1 store | N = 354  Low income, 80% female, children under 18 years, | 7 months (3 months baseline, 4 months post-intervention | Loyalty card transactions | N/A | Weekly sales ($) eligible FV | Linear regression | Intervention arm increased purchases of all FV (15%), fresh (18%), vegetables (20%), but no increase for fruit, little or negative effect for frozen and canned FV, vs control  SNAP participants increased FV by 45%, vs 11% non-SNAP 53% increase in fresh, vs 13% non-SNAP |
| Targeted coupons | Guan et al. (2018) | | Quasi-experimental | Influence of individually-targeted coupons on purchasing patterns for less healthful and more healthful products | USA  5 stores from same supermarket chain | N = 2,500  Convenience sample  Demographic characteristics not described | 2 years (2003 – 2005) | Loyalty card transactions collected by EPOS provider Dunnhumby | USDA Quarterly Food-At-Home Price Database (QFAHPB) used to categorise products | Weekly purchases (units) of 12 ‘healthful’ and ‘less healthful’ categories | Difference in difference analysis  ANOVA | Weekly purchases increased from pre-post intervention periods for both exposed and unexposed. Exposed purchased 5.06 units more p<0.001  Positive difference in difference for all 12 groups, greatest for less healthful; convenience foods +1.17 units, lowest = nuts +0.03 units |
| **Community interventions** | | | | | | | | | | | | |
| Shop Smart 4 Health | | Ball, Kylie et al. (2016) | RCT | Cost-effectiveness of skill-building to promote purchase & consumption of fruit & veg | Australia, Coles stores  Number of stores not stated | Low income women, regular shoppers at stores in deprived areas | 12 months (6 months intervention + 6 months follow up) | Loyalty card transactions  Self-reported portions/day, FFQ for past 6 months | N/A | Purchases of veg & fruit (g/hh/wk), consumption (serves/day) | Generalised Estimating Equations, Mediation analysis & cost-consequence analysis (broad societal perspective) | 0.49 (CI 0.25, 0.72) portions more veg consumed immediately after, at 6-months +0.28 serves/day (CI 0.04, 0.52), ICER = $3.10/extra veg serve/person/day |
| Supermarket Healthy Options Project (SHOP) | | Eyles, H. et al. (2010) | Feasibility study | Develop culturally tailored nutrition education resources | New Zealand  6 stores | N = 551  Maori = 123, Pacific = 52, European /other = 346 | 3 months baseline purchase data used to inform design of materials | Store self-scan transaction data  Australian Heart Foundation Tick nutrient profile to identify ‘healthier’ products | Supermarket Food & Nutrition Database (SFND);  Manufactured Food Database, brand websites, back of pack & NZ national food tables | Feasibility of applying nutrient profiling | N/A | 1814 (60%) products classified as ‘healthier’  Food & nutrient database successfully linked to 3 months transaction data  Monthly reports automatically generated tailored shopping lists based on purchases |
| 1% or Less campaign | | Reger, B. et al. (1998) | Controlled community intervention | Effect of community education + mass media encouraging low fat milk consumption to reduce saturated fat intake | West Virginia, USA  2 intervention communities, 1 control (convenience sampled) | N = 25,000 in intervention communities  N = 34,000 in control community | 3 months (February – April 1995) | Monthly supermarket sales, & hh intake from telephone interviews | N/A | Mean sales milk (gallons)/supermarket/month (whole milk, 2% fat, 1%, ½% & skim)  Market share by category (% total gallons)  Self-reported consumption, % high/low fat milk drinkers and switching | Repeated measures ANOVA  2-tailed t-tests and F-tests at 5% significance level | Sales increased by 16%, low fat share up 23%. Greatest increase for 1% milk. Decrease in high fat milk, similar in control  38% respondents switched to low-fat, no difference by group, or individual characteristics. |
|  |  | Reger, Bill et al. (1999) |  | Effect of mass media to change milk consumption | Wheeling, West Virginia USA | Population 35,000 | 6 weeks (February – March 1996) |  |  |  |  | Low fat milk share increased17%, high fat decreased 13%. No difference in overall milk sales |
|  |  | Reger, B. et al. (2000) |  | Advertising vs PR & community education | Rural West Virginia, USA | PR + education (n = 34,000), Advertising (n = 18,000), Control (n = 14,000) | 8 weeks in winter 1997 |  |  |  |  | PR + education: 19.6% switched to low-fat, 12.8% advertising, 6.8% control. |
| Towards a Healthy Diet | | Dunt et al. (1999) | Quasi-experimental | Promote healthy diet policy changes in schools, health services, restaurants etc. | Victoria Australia  2 cities; 5 intervention stores, 4 control | N = 1137 completed panel questionnaires  N = 703 completed cross-sectional survey | 2 years (October 1991 – October 1993) | Monthly supermarket sales  Panel and cross-sectional surveys | N/A | Sales volume of milk & table spreads /supermarket/month  Self-reported opinion, dietary behaviour, cognition about healthy diet etc. | Mann-Whitney U-test for survey evaluation  Method not stated for sales data | Modest positive changes in individuals - only significant group difference between = decrease in takeaway foods in intervention.  No downward trend in unhealthy purchases. |
| **In-store choice architecture** | | | | | | | | | | | | |
| Project Sol | Winkler et al. (2016) | | Non-randomised intervention | Examine customer attitudes & sales effects of healthy checkout supermarket intervention | Denmark  4 chains owned by Coop group 28 stores (4 intervention, 12 control, 12 other areas) | Customers of different supermarket chains. Customer demographics unknown | 5 months, 4 weeks intervention | Weekly store sales | N/A | Weekly store sales (revenue); all foods | Linear mixed models | Positive effect on carrot snack pack sales, but no other healthy snacks or fruit.  Confectionary sales unaffected. |
|  | Toft et al. (2017) | | Non-randomised cluster intervention | Effect of improved shelf-space with & without 20% price discount for fruit & veg | Denmark  5 discount stores (2 intervention, 3 control) in 2 regions | Customer demographics unknown | 5 months (1-month pre-, 3 months intervention, 1-month post) | Sales from Netto stores  Sales from other intervention area supermarkets |  | Weekly store sales (units); fruit & veg (fresh, frozen, canned and dried); Index relative to previous year | Multi-level regression analysis | Shelf-space + price increased fresh by 22%, organic fresh by 12.1%, total by 15.3% No effect for shelf-space only  No unhealthy substitution effects |
| Omega-3 podcasts | Bangia et al. (2017) | | Non-randomised experiment | Impact of store podcast tour intervention on omega-3-rich foods | USA, New Jersey  20 stores in middle- & upper-middle class areas | N = 173  Loyalty customers who listened to n-3 podcasts during a main shop & shopped at least once/month during study period | 12 months  (6 months pre-, 6 months post-intervention, 1 day) | Daily store loyalty card data | N/A | Sales of targeted n-3-rich foods (units/participant/ month by food type & category) | Pearson’s correlation (intention & purchase)  Wilcoxon signed-rank – pre-post differences by gender, SNAP participation & food type/ category  Kruskal-Wallis 1-way ANOVA – pre-post differences by race or education | 59% of shoppers increased n-3-rich purchases. Mean items significantly increased from 0.2 (SD 0.7) to 3.6 (SD 5.1)  Increase in fortified foods greater for women than men (+2.68 items). No other demographic differences  No relationship between intention & purchases |
| Keyhole campaign | Mork et al. (2017) | | Before and after | Impact of Keyhole awareness campaign on purchases of Keyhole-labelled products | Denmark  6 stores from 3 chains (2 regular, one discount chain) | Target = men >35-years with low education  Data for all customers, no demographic information | 9 weeks  (3 weeks pre- and 3 weeks post-intervention) | Transaction data  In store observation & researcher interviews | N/A | Daily store sales of 10 food categories – turnover (volume & value) by category & Keyhole status | Multi-level logistic regression | Odds of purchasing Keyhole labelled products rose by 20% in standard stores, 10% decrease in discount stores.  Purchase more likely linked to health motives among participants with short education. |
| POP intervention | Freedman and Connors (2010) | | Quasi-experimental pilot study | Effect of shelf tags to promote healthy choices | USA  1 on-campus convenience store at large urban university | Number unknown  No customer demographics  University students; 23% Asian, 16% Hispanic, 29% White, 32% Other | 11 weeks (6 weeks fall 2008 semester + 5 weeks spring 2009) | Sales from computerised cash register | On pack nutritional information used to allocate tags indicating healthy food choice | Sales of tagged (healthy) & untagged (unhealthy) foods in 4 categories; cereal, soup, crackers, bread | Mann-Whitney U test at 95% confidence level | Increased sales of tagged items during intervention for cereal, soup & crackers but decrease for bread.  Overall sales of tagged items increased 3.6% (SD 1.6%) P = 0.082 |
| Shopper marketing intervention | Payne et al. (2015) | | Non-randomised experiment | Efficacy of shopper marketing on produce demand, store profits & shopper budgets | El Paso, Texas, USA  4 stores (3 intervention, 1 control) | No customer demographics  Area-level demographics;95% Hispanic, 53% female, Mean age ~30 years | Pilot = 14 days, main study = 28 days intervention + baseline & follow up  2012 - 2013 | Store-level aggregated sales data | N/A | Total produce spend/person/ day, proportion of baseline & total expenditure (%) | T-test | Pilot: Significant increase in produce spend in intervention (+16%), not control (+4%). No change in overall spend.  Main study: Both stores increased spend (+12.4% & +7.5%). proportion of total spend increased (+13.3% + 8.5%). No change in overall spend. |
| Manger Top intervention | Gamburzew et al. (2016) | | Difference in differences | Social marketing to draw attention to inexpensive healthy foods | Marseilles, France  2 disadvantaged areas  4 discount stores (DIA) | Purchase data N = 6,625 loyalty card holders  Survey subset (N = 116); 78% female, 16% food insecure, 31% aged >60years | 18 months (January 2013 – June 2014), 6-month intervention (January- June 2014) | Loyalty card transactions  In-depth survey | French food composition database | Contribution of inexpensive healthy foods to total food spend (%) & spend by category | Generalised linear model  Chi-squared test  Fisher tests  One-way ANOVA | Contribution to total food spend ~20% for both groups, increased in 2014.  No significant difference overall but greater increase in fruit, veg & starches for intervention stores |
| Eat Right ‘N’ Live Well! | Surkan et al. (2016) | | Non-randomised intervention | Multifaceted supermarket intervention promoting healthier alternatives to commonly purchased foods | Baltimore, USA  2 stores in low-income African-American areas | Customer number unknown.  Area demographics, 76% African-American, 20% unemployed, 33% single-parent hhs | 3 years  (July – October for 2010, 2011 and 2012) | Store-level aggregated sales | N/A | Number of items (units) sold, absolute & % differences in sales | Difference-in-difference analysis | Higher growth in sales of promoted foods in intervention store (+10.8%) vs control (+9.3%)  Moderate success, not uniform across food categories |
| Guiding Stars | Hobin et al. (2017) | | Natural experiment | Impact of Guiding Stars shelf labelling on nutritional quality of food purchases | Ontario, Canada  126 stores, 3 supermarket chains owned by the same company | Customer number unknown, 145 million transactions  783 exit interview participants; 75.0% female, 47.6% overweight/obese, 83.3% White | 1 year  (June 2012 – July 2013)  Guiding Stars in 1 chain in August 2012 | Aggregated supermarket transactions/day | Guiding Stars Licensing Co food and nutrient database – UPC-level, >55,000 products | Change in stars /product & /serve  Change in calories & nutrients /serve  Quantity of products /transaction, price/product, store revenue | Difference-in-difference analysis  Regression analysis, controlling for seasonality | Significant increase in mean star rating (+1.4%), share of 1- and 3-star products (+2% & 1.9%), decline in 0-2-star products (-0.7% & -1.9%). 3.5% & 1.5% decrease in trans-fat & sugar, & 0.6% & 4.5% increase in fibre & omega-3  Number of products, price /product & store revenue increased. Effect varied by category. |
| Portion intervention | Vandenbroele et al. (2018) | | Non-randomised controlled experiment | Effect of 2 additional smaller portion options on portions purchased meat sausages | Belgium  9 stores from large European retailer (1 intervention, 8 control) | N = 161 Loyalty card who customers bought target product  59% female. No demographics for baseline or control | 1 month | Supermarket aggregated & individual-level sales | N/A | Unit sales & volume (kg) /store & /individual for target product (3 portions) & meat category | 2-way ANOVA – before & during, experiment vs control stores  Control for backfire (purchasing multiple) & compensation within meat category | Slightly higher sales of two new smaller portions (52% combined) vs original (48%). Reduced total sales volume (kg). Small portion customers bought significantly less (kg) (M = 0.33, SD 1.90) than large customers (M = 0.49, SD = 1.91).  No compensation |
| Make it Fresh for Less | Moran et al. (2019) | | Quasi-experimental and RCT | Effect of healthful low-cost meal bundles (quasi-experimental) and electronic reminders (RCT) on purchases of healthy meal bundle items | USA  2 stores from large supermarket chain in Portland, Maine | N = 238 in RCT, intervention = 126, control = 112  81% female, 90% non-Hispanic White, 25% used SNAP benefits | 13 months  40 weeks baseline, 16 weeks intervention | Store sales and loyalty card transactions | N/A | Sales ($) of meal bundle items, by transaction, or monthly by store | Linear regression | No effect of electronic reminders on purchases of meal bundle items.  No significant increase in sales of bundled items in intervention store vs control. |
| Checkout nudges | Kroese et al. (2016) | | Non-randomised controlled experiment | Investigate effect of a food repositioning nudge on healthy food choice | Netherlands  3 kiosks (small convenience stores) at train station | N = 91 participated in exit interviews  52% male, mean age 39 yrs (SD 15.75 yrs)  Demographics of overall customer-base unknown | 2 weeks (1 week baseline, 1 week nudge intervention) | Store-level daily sales (items) | N/A | Daily sales of nudged ‘healthier’ snacks (number of units) | ANCOVA | Significant difference in mean daily number of nudged items sold between stores  Control = 23 items Nudge = 41 (p=0.00), Nudge + disclosure = 35 (p=0.02)  No difference between nudge and nudge + disclosure (p=0.17)  No difference in sales of non-nudged items |
|  | Van Gestel et al. (2018) | | Longitudinal |  | Netherlands  1 kiosk (small convenience store) at train station | N = 186 participated in exit interviews  57% male, mean age 38 yrs (SD 17 yrs)  Demographics of overall customer-base unknown | 8 weeks (4 weeks baseline, 4 weeks nudge intervention) | Store-level daily sales  Individual-level purchases evaluated in exit interviews | N/A | Daily sales of selected healthy products as a proportion of total food sales | ANOVA | 179 food products sold.  Sales of total and healthy food products higher during baseline.  Proportion of targeted healthy foods sold higher in nudge phase (mean = 6.3% SD 1.4) than baseline (mean = 4.3% SD 0.9)  Effect maintained over 4-week nudge period |
| University store choice architecture | Walmsley et al. (2018) | | Natural experiment | Effect of choice architecture intervention, re-arrangement of produce to increase the accessibility of fruit and vegetables | UK, Warwick  1 store on University Campus | Number unknown  University students (26,000) and staff | 5.5 years, excluding non-termtime weeks  90 weeks baseline, 40 weeks intervention A, 40 weeks intervention B | Store level sales data aggregated weekly | N/A | Fruit and vegetable (FV) sales (units and monetary spend) as a proportion of total food sales | Retrospective interrupted timeseries modelling  Dynamic regression with Auto Regressive Integrated Moving Average (ARIMA) | Significant increase in proportion of FV for intervention A. Non-significant increase for intervention B.  Overall downward trend in proportion of sales that were FV over the 5.5 year study period. |
| Online supermarket | Martinez et al. (2018) | | Mixed methods  RCT | Examine impact of pilot for online grocer to accept Electronic Benefit Transfer (EBT) Cards | USA  Low-income neighbourhood in the Bronx | N = 148 included in baseline data  1/3 = EBT users  N = 348 recruited to RCT | 9 months  September 2012 – June 2013 | Online grocery transactions | N/A | Average spend per order (% of purchase) on 5 food groups; fruit, veg, dairy, sweets, salty snacks | Mann-Whitney U test | EBT orders spent more on sweets (10.8% vs 4.9% non-EBT) and salty snacks (2.2% of purchase, vs 1.1%), slightly less on fruit (6.3% vs 8.7%) . No significant difference for spend on veg (11.5% vs 14.2%) and dairy (both groups 8.5%) |
| **Comparison with dietary intake** | | | | | | | | | | | | |
| Supermarket Healthy Options Project (SHOP) | Hamilton et al. (2007) | | Observational | Compare supermarket nutrient availability with national consumption & expenditure surveys | New Zealand  1 store | N = 882 customers eligible for SHOP pilot RCT  Age (µ) 38yrs, 73% female | 12 months (February 2003 – January 2005) | Store self-scan (Shop ‘N Go) transactions  National consumption & expenditure surveys | Supermarket Food and Nutrition Database (SFND);  Manufactured Food Database, brand websites, back of pack & NZ food tables | Proportion sales volume (units) & expenditure (%) by food category/hh  macro-nutrients % energy  Contribution of food groups to macro-nutrients | Difference between supermarket and survey data | Similar to survey for CHO, total fat & saturates, protein lower. Less comparable with children’s survey (supermarket lower CHO, similar protein & saturates, & higher total fat).  Expenditure similar for most foods, supermarkets lower for sweet foods & beverages |
|  | Eyles, Helen et al. (2010) | | Observational | Household electronic sales data vs individual nutrient intakes from 24-hr recalls | New Zealand  6 stores | N = 49 participants from SHOP RCT  Age (µ) 48yrs, 84% female, 53% university/ tertiary qualifications | 3 months (Nov 2004 – Jan 2005) | Self-scan (Shop ‘N Go) transactions – coded to (SFND) 3000 top-selling foods  4 non-consecutive dietary recalls – coded to national food composition database (>2600 foods) |  | Household energy (E) & energy-adjusted macronutrients  Energy density (ED) (beverages & non-beverages) | Spearman correlation coefficients  Paired t-tests  2-sided at 5% significance level | Moderate correlation: Saturates (%E) R^2^ = 0.54**, CHO (%E) R^2^ = 0.48**, Protein (%E) R^2^ = 0.44**, Fat (%E) R^2^ = 0.34, Sugar (%E) R^2^ = 0.33, ED nonbev (kcal/oz) R^2^ = 0.37, ED bev (kcal/oz) R^2^ = 0.09, Sodium (kcal/oz) R^2^ = 0.06  No difference for saturates & total fat. Significant for; CHO +3%, Protein -4%, Sugar -2.1oz/kcal, Sodium -122.84, Oz/kcal |
| **Population dietary surveillance** | | | | | | | | | | | | |
| USDA report | Frazao and Allshouse (1996) | | Observational | Report the size & growth of USA nutritionally improved foods market | USA  3,000 supermarkets | No demographic information | 5 years (1989 – 1993) | Store sales from supermarkets with annual revenue >$2 million | N/A | Volume sales, dollar sales, volume & dollar share of nutritionally improved products | Descriptive only, no statistical tests | Nutritionally improved cost more. Availability increased.  Volume sales and dollar sales increased from 1989 to 1993 |
| Columbus supermarket study | Jones (1997) | | Observational | Difference in price elasticity for high & low-income customers | USA  7 stores | No customer demographic information. Supermarkets classified as high or low-income area based on census tract | 2 x 54-weeks; 1990 - 1991  1993 - 1995 | Weekly purchases by store | N/A | Price/ ounce (ratio of group sales)  Elasticity of demand | Time-series cross-section regression model, error components model | Elasticity high for breakfast cereal, low for carbohydrates. Differences by income group for cereals. Lower income pay less per ounce & twice as price sensitive |
| Bread purchases | Revoredo-Giha et al. (2009) | | Observational | Effect of price changes on consumption of different bread types in Scotland | Scotland; 3 TV regions (Borders, Central, North)  Major UK supermarket | Number & demographics not reported.  3 geodemographic groups - proxy for affluence | 2 years (Oct 2006 – Sept 2008) | Scanner data from loyalty customers | N/A | Regional & SES weekly sales premium & non-premium brown & white bread (g/person /day), (£/g) | 3 demand models: Rotterdam demand system, Static LA/AIDS, Dynamic LA/AIDS | Brown & white bread quite price elastic - consumption reduces when prices increase, particularly brown bread.  No difference in price elasticity by region or socioeconomic group |
| Finnish supermarket study | Närhinen et al. (1998) | | Cross-sectional | Variation in daily sales, usefulness of supermarket data for monitoring & evaluating shopping behaviour | Finland  1 store in Mikkeli, town with ~30,000 inhabitants | All customers, no demographic information | 2 months; May 1996, September 1996 | Daily cash register sales aggregated weekly & monthly | N/A | Direct & proportional sales, 79 healthier & reference products, 17 categories (number, kg, price/kg) | Mean, standard deviation, coefficient of variation | Proportional more stable than direct sales.  Variation similar for 1 week/1 month sales, greater daily variation – sales of milk & yoghurt higher on Fridays |
|  | Närhinen et al. (1999) | |  | How well does supermarket sales data reflect regional diet differences | Finland  8 Prisma stores, 6 cities |  | 1 month, September 1997 | Cash registers & 3 yrs health survey results (1995 – 1997) |  | Proportional sales milk, sour milk, fats, oils  Mean salt & fat % & proportion saturated:total fat | Chi-squared | Regional differences in sales & survey data, high similarity (value not stated). Reported use of non-fat milk higher than actual sales. |
| Dalby CVD campaign | Radimer and Harvey (1998) | | Cross-sectional | Validity of self-reported use of reduced fat & salt foods | Australia  Remote community Dalby, 1 supermarket store | 453 questionnaire respondents, no demographic information | 1 year, 1992 - 1993 | Sales data & FFQ 1 yr  Milk deliveries data; 2 months 1993 | N/A | % reporting use of reduced fat & salt foods  Sales reduced fat & salt foods (adjusted for national adult milk consumption)  Milk deliveries | Store sales within 91% of survey data (Y/N) | Reported consumption reduced fat & salt foods greater than sales & deliveries suggest  Largest difference for reduced salt bread & soup, smallest for butter & margarine. |
| Study of Danish wine and beer drinkers | Johansen et al. (2006) | | Observational | Investigate diet patterns of wine & beer buyers | Denmark  98 outlets; 2 large chains owned by Dansk Supermarked; 16 Bilka, 82 Fotex | Customers of Bilka & Fotex, likely to over-represent middle income.  No customer demographic information. | 6 months (September 2002 – February 2003) | 3.5 million transactions | N/A | Daily purchases of 40 food categories/ customer | Correspondence analysis & logistic regression | Wine buyers spent more & bought more items. More likely to follow Mediterranean diet – olives, fruit & veg, poultry, oil, & low-fat cheese, milk, meat  Beer buyers follow traditional diet – ready meals, sugar, cold cuts, chips, pork, butter/margarine, sausages & lamb |
| Philadelphia supermarket | Phipps, Etienne J. et al. (2013) | | Observational | Investigate predictors of fresh fruit & veg purchases in low income population | Philadelphia, USA  1 store, low-income minority ethnic community | 30 low income loyalty card households, at least 1 child  Primary household shopper; 90% female, 87% African- American, Mean age 42yrs (±14) | 3 months,  April 1 – June 30 2010 | Loyalty card point of sale data | N/A | Primary = servings fresh fruit & veg/hh/week  Secondary = total fresh produce expenditure/hh/week | Bivariate & multivariable Poisson regression with log link | Controlling for household size, average servings +50-60% for each extra child (P=0.008), +10% for every year in age range of children (P=0.04)  Mean servings/week = 4.0 (±2.9)  No association with poverty, income, benefits, age or education of primary shopper |
|  | Phipps, E. J. et al. (2014) | |  | Impact of price discount on purchases of high-calorie foods (HCF) & low-calorie foods (LCF) |  | 82 primary household shoppers with loyalty card.  Adults ≥1 child, primarily female African-American | 65 weeks; October 2012 – November 2013 |  | Not stated | Weekly household sales of HCF & LCF, ratio odds of purchase, sale vs full price  % cost saving/day | Fixed effects logistic regression for ratio of odds  Bivariate & multivariate fixed effects generalised linear regression | Odds of buying on sale vs full price higher for grain-based snacks, sweet snacks & SSBs (OR = 6.6, 5.9, 2.6 respectfully) all P<0.001. Not for savoury snacks or LCFs. |
| Swiss loyalty card study | Hauser et al. (2013) | | Observational | Investigate how food-related values & attitudes influence purchases by category | Switzerland, 2 regions (German & French-speaking), 1 supermarket chain | 851 loyalty card holders | Purchases 1 year prior to survey | Purchase data from loyalty card holders  Values & attitudes survey | N/A | Annual hh expenditure/ category (% total food expenditure) | Confirmatory factor analysis  Structural equation modelling | Moderate correlation between values & purchases. Fruit & veg associated with sustainability & health values. Fresh convenience & ready-to-eat positively correlated with convenience, negatively with conviviality & health. |
| Casino study | Hansel et al. (2015) | | Observational | Relationship between purchases of alcoholic beverages & food | France, urban & rural areas  Casino supermarkets | 196,000 loyalty card holders, regular shoppers  No demographic information | 1 year (September 2010 – September 2011) | Purchase data from loyalty card holders | N/A | % of hh annual budget for alcohol, healthy & unhealthy food, ratio of total | k-means clustering by alcohol purchase  chi-squared | Wine purchasers spent higher proportion of their budget on healthy foods vs beer or non-alcohol buyers.  Non-alcohol buyers - lower total spend. |
| Healthy Food Program | Sturm et al. (2016) | | Observational | Relationship between seasonal food purchases & BMI | South Africa | 25% rebate (N = 400,000 households)  BMI data for ~ 500,000 individuals | 4 years (2009 – 2013) | Purchases from eligible supermarkets using health insurance credit card  Health Risk Assessment Survey (HRA) | N/A | Monthly spend/category/hh : total spend monthly/hh | Multiple regression analysis at household (purchases) & individual level (BMI) | 13% expenditure fruit & veg, 9% other healthy foods. December; 41% higher purchases of less desirable foods, lower fruit & veg purchases (vs January)  Annual weight gain +0.13 BMI units (men +0.43kg, women +0.3kg). Christmas weight gain ~60-70% of annual (men +0.1 BMI units, +0.35kg, women +0.8 BMI units, +0.2kg) |
| Healthy Trolley Index | Taylor et al. (2015) | | Observational | Healthy Trolley Index (HETI) to estimate diet quality & compare purchases with dietary guidelines | Australia  Staff from large retailer corporate office wellness scheme, | 964 loyalty card holders; mean age 37.6 yrs (SD 9.3), 56% female, mean BMI 27.9 (SD 6.6), 23% overweight, 28% obese, 15% live alone | 1 month (April – May 2014) | Purchase data from loyalty card holders | N/A | HETI (/100) high = compliance Australian Guide to Healthy Eating (AGHE)  Expenditure/ HETI group, proportion total food & drink | One-way ANOVA & Bonferroni post-hoc tests  Chi-squared difference in shopping frequency by weight | Average HETI = 58.8 (SD 10.9), higher for males & normal BMI  62.7% met meat & alternatives guidance, compliance poor for grains (0.2%), discretionary (1.8%) & veg (5%). |
| SNAP monthly purchases | Franckle et al. (2019) | | Secondary analysis of RCTs | Examine purchase fluctuations over the SNAP benefit month, for SNAP households and non-SNAP households | USA  2 same chain supermarkets in low-income communities in Maine | N=950 loyalty card holders who participated in RCTs  84% female, 94% White non-Hispanic | Up to 8 months of data | Daily purchases aggregated by week and by month, from loyalty card issued for RCT | N/A | Mean spend ($) per transaction on all foods and for selected categories in first 2 weeks, and last 2 weeks of the month after SNAP benefits issued | Difference-in-difference | 37% decline for SNAP (all categories), 3% for non-SNAP (only red meat and poultry)  SNAP decline by category; veg -25%, fruit -27%, SSBs -30%, red meat - 37%, convenience -40%, poultry - 48% |
| LoCard | Nevalainen et al. (2018) | | Longitudinal | Address potential and challenges of loyalty card data for health research. | Finland, 1 grocery chain | N = 14,595 households  >13 million transactions | 1 year (1 January – 31 December 2016) | Loyalty card data | N/A | Total annual grocery expenditure (Euros)  Participant and purchasing profiles | Descriptive statistics, linear regression, logistic regression, inverse probability weighting to adjust for non-participation bias | Gender and age are significant determinants of expenditure (peak at middle age).  Men's expenditure greater than women's.  Top 10 selling product groups included 'beer and cigarettes'. Fridays and Saturdays = most active purchase days. National holidays preceded by peak in spend |
|  | Uusitalo et al. (2019) | | Longitudinal | analyse alcohol purchase patterns on the level of individual shopping occasions. |  | N = 13,274 households |  |  |  | Total expenditure (Euros) & proportion of total basket expenditure on alcohol (beer, cider, non-alcoholic equivalents), cigarettes & food groups | K-means cluster analysis based on alcohol purchases  Linear mixed models to assess difference in means between clusters | 8 clusters, most common = no alcohol (86.1%) (reference)  Beer buyers mostly men, and older.  More alcohol associated with more food, especially meat, soft drinks, cheese, sweet foods, fat, breads, ready to eat. But lower fruit expenditure. |
| **Methodological** | | | | | | | | | | |  |  |
| Informatics feasibility study | Brinkerhoff et al. (2011) | | Feasibility study | Feasibility of linking point-of-sale data to USDA-SR nutrient database | USA, Intermountain West  Large supermarket chain | 32,785 customers  2,009,533 de-identified sales items  No demographic information | 2 weeks, August 2007 | Individual customer purchase data | United States Department for Agriculture National Nutrient Database for Standard Reference (USDA-SR)  >7,500 food items, 24 food groups | Match-rate (%) between product in supermarket sales database and USDA-SR | No statistical analysis methods  String-matching & fuzzy matching at the food item level, manual matching | 3-tier organisational hierarchy (Department 🡪 Commodity 🡪 Sub-commodity)  70% sub-commodities mapped to SR food items (complete nutritional data), 100% of sub-commodities mapped to SR food groups |
| qDIET | Chidambaram et al. (2013) | | Feasibility study | qDIET method - automated & self-sustaining linkage between retail data & USDA databases to calculate HEI | USA, Salt Lake City  Large national grocery retailer | 50 households who reported >75% food intake from retail stores  No demographic information | 12 months (February 2007 – April 2008) | Household purchase data for loyalty card holders | Food & Nutrition Database for Dietary Studies (FNDDS), MyPyramid Equivalents Database (MPED), Google Shopping API & Factual.com API | Match-rate (%) between product in supermarket sales database & API product data | Non-parametric Kolmogorov-Smirnov empirical distribution function two-sample test | No significant difference in HEI scores distribution between retail households & NHANES  30.7% of the 12,332 products matched by Google API, 71.5% matched by Factual |
| FPED | Tran et al. (2017) | | Feasibility study | Systematic food quality monitoring by automated mapping to USDA Food Patterns Equivalent Database (FPDB) | USA, 4 geographic regions  1 grocery chain | 144,000 households  190 million transactions  92,062 distinct grocery items | 15 months (January 2012 – March 2013) | Household purchase data | USDA databases; Food & Nutrient Database for Dietary Studies (FNDDS), Food Pattern Equivalents Database (FPED) | Match-rate (%) between product in supermarket sales database & FPED | Confidence coefficient for similarity between grocery description & food FPED categories | Match-rate per category between 77% - 100%  Mappings more complex for mixed dishes & ethnic foods – yet to be verified |

### References S4.

Alvarado, M., Unwin, N., Sharp, S.J., Hambleton, I., Murphy, M.M., Samuels, T.A., Suhrcke, M. and Adams, J. 2019. Assessing the impact of the Barbados sugar-sweetened beverage tax on beverage sales: an observational study. *International Journal of Behavioral Nutrition & Physical Activity.* **16**(1), p13.

An, R. 2014. Eating better for less: Effectiveness of financial incentives in modifying dietary and grocery shopping behavior. *Dissertation Abstracts International: Section B: The Sciences and Engineering.* **74**(9-B(E)), pp.No-Specified.

Andreyeva, T. and Luedicke, J. 2013. Federal food package revisions: effects on purchases of whole-grain products. *American Journal of Preventive Medicine.* **45**(4), pp.422-429.

Andreyeva, T. and Luedicke, J. 2015. Incentivizing fruit and vegetable purchases among participants in the special supplemental nutrition program for women, infants, and children. *Public Health Nutrition.* **18**(1), pp.33-41.

Andreyeva, T., Luedicke, J., Henderson, K.E. and Schwartz, M.B. 2014. The positive effects of the revised milk and cheese allowances in the special supplemental nutrition program for women, infants, and children. *Journal of the Academy of Nutrition and Dietetics.* **114**(4), pp.622-630.

Andreyeva, T., Luedicke, J., Henderson, K.E. and Tripp, A.S. 2012. Grocery store beverage choices by participants in federal food assistance and nutrition programs. *American Journal of Preventive Medicine.* **43**(4), pp.411-418.

Andreyeva, T., Luedicke, J., Tripp, A.S. and Henderson, K.E. 2013. Effects of reduced juice allowances in food packages for the women, infants, and children program. *Pediatrics.* **131**(5), pp.919-927.

Balasubramanian, S.K. and Cole, C. 2002. Consumers' search and use of nutrition information: The challenge and promise of the Nutrition Labeling and Education Act. *Journal of Marketing.* **66**(3), pp.112-127.

Ball, K., McNaughton, S.A., Le, H.N., Abbott, G., Stephens, L.D. and Crawford, D.A. 2016. ShopSmart 4 Health: results of a randomized controlled trial of a behavioral intervention promoting fruit and vegetable consumption among socioeconomically disadvantaged women. *The American journal of clinical nutrition.* **104**(2), pp.436-445.

Ball, K., McNaughton, S.A., Le, H.N.D., Gold, L., Ni Mhurchu, C., Abbott, G., Pollard, C. and Crawford, D. 2015. Influence of price discounts and skill-building strategies on purchase and consumption of healthy food and beverages: Outcomes of the supermarket healthy eating for life randomized controlled trial. *American Journal of Clinical Nutrition.* **101**(5), pp.1055-1064.

Banerjee, T. and Nayak, A. 2018. Believe it or not: Health education works. *Obesity Research and Clinical Practice.* **12**(1), pp.116-124.

Bangia, D., Shaffner, D.W. and Palmer-Keenan, D.M. 2017. A Point-of-Purchase Intervention Using Grocery Store Tour Podcasts About Omega-3s Increases Long-Term Purchases of Omega-3-Rich Food Items. *Journal of nutrition education and behavior.* **49**(6), pp.475-480.

Bartlett, S., Klerman, J., Olsha, L., Logan, C., Blocklin, M., Beaurgard, M., Enver, A. 2014. *Evaluation of the Healthy Incentives Pilot (HIP) Final Report.*

Bernales-Korins, M., Ang, I.Y.H., Khan, S. and Geliebter, A. 2017. Psychosocial influences on fruit and vegetable intake following a NYC supermarket discount. *Obesity.* **25**(8), pp.1321-1328.

Blakely, T., Mhurchu, C.N., Jiang, Y., Matoe, L., Funaki-Tahifote, M., Eyles, H.C., Foster, R.H., McKenzie, S. and Rodgers, A. 2011. Do effects of price discounts and nutrition education on food purchases vary by ethnicity, income and education? Results from a randomised, controlled trial. *Journal of Epidemiology and Community Health.* **65**(10), pp.902-908.

Brimblecombe, J., Ferguson, M., Chatfield, M.D., Liberato, S.C., Gunther, A., Ball, K., Moodie, M., Miles, E., Magnus, A., Mhurchu, C.N., Leach, A.J., Bailie, R. and collaborative, S.R.r. 2017. Effect of a price discount and consumer education strategy on food and beverage purchases in remote Indigenous Australia: a stepped-wedge randomised controlled trial. *The Lancet. Public health.* **2**(2), pp.e82-e95.

Brinkerhoff, K.M., Brewster, P.J., Clark, E.B., Jordan, K.C., Cummins, M.R. and Hurdle, J.F. 2011. Linking Supermarket Sales Data To Nutritional Information: An Informatics Feasibility Study. *AMIA Annual Symposium Proceedings.* **2011**, pp.598-606.

Brunello, G., Paola, M.d. and Labartino, G. 2012. More apples less chips? The effect of school fruit schemes on the consumption of junk food. *IZA Discussion Papers - Forschungsinstitut zur Zukunft der Arbeit.* (6496), pp.23-pp.

Brunello, G., Paola, M.d. and Labartino, G. 2014. More apples fewer chips? The effect of school fruit schemes on the consumption of junk food. *Health Policy.* **118**(1), pp.114-126.

Chidambaram, V., Brewster, P.J., Jordan, K.C. and Hurdle, J.F. 2013. qDIET: toward an automated, self-sustaining knowledge base to facilitate linking point-of-sale grocery items to nutritional content. *AMIA ... Annual Symposium proceedings. AMIA Symposium.* **2013**, pp.224-233.

Dunt, D., Day, N. and Pirkis, J. 1999. Evaluation of a community-based health promotion program supporting public policy initiatives for a healthy diet. *Health Promotion International.* **14**(4), pp.317-327.

Eyles, H., Jiang, Y. and Ni Mhurchu, C. 2010. Use of household supermarket sales data to estimate nutrient intakes: a comparison with repeat 24-hour dietary recalls. *Journal of the American Dietetic Association.* **110**(1), pp.106-110.

Eyles, H., Rodgers, A. and Ni Mhurchu, C. 2010. Use of electronic sales data to tailor nutrition education resources for an ethnically diverse population. *Journal of Human Nutrition and Dietetics.* **23**(1), pp.38-47.

Ferguson, M., O'Dea, K., Holden, S., Miles, E. and Brimblecombe, J. 2017. Food and beverage price discounts to improve health in remote Aboriginal communities: mixed method evaluation of a natural experiment. *Australian and New Zealand journal of public health.* **41**(1), pp.32-37.

Franckle, R.L., Levy, D.E., Macias-Navarro, L., Rimm, E.B. and Thorndike, A.N. 2018. Traffic-light labels and financial incentives to reduce sugar-sweetened beverage purchases by low-income Latino families: a randomized controlled trial. *Public Health Nutrition.* **21**(8), pp.1426-1434.

Franckle, R.L., Thorndike, A.N., Moran, A.J., Hou, T., Blue, D., Greene, J.C., Bleich, S.N., Block, J.P., Polacsek, M. and Rimm, E.B. 2019. Supermarket Purchases Over the Supplemental Nutrition Assistance Program Benefit Month: A Comparison Between Participants and Nonparticipants. *American Journal of Preventive Medicine.* **57**(6), pp.800-807.

Frazao, E. and Allshouse, J.E. 1996. Size and growth of the nutritionally improved foods market. *Agriculture Information Bulletin - United States Department of Agriculture.* (723), pp.iv-pp.

Freedman, M.R. and Connors, R. 2010. Point-of-Purchase Nutrition Information Influences Food-Purchasing Behaviors of College Students: A Pilot Study. *Journal of the American Dietetic Association.* **110**(8), pp.1222-1226.

Gamburzew, A., Darcel, N., Gazan, R., Dubois, C., Maillot, M., Tome, D., Raffin, S. and Darmon, N. 2016. In-store marketing of inexpensive foods with good nutritional quality in disadvantaged neighborhoods: Increased awareness, understanding, and purchasing. *The International Journal of Behavioral Nutrition and Physical Activity.* **13**.

Geliebter, A., Ang, I.Y.H., Bernales-Korins, M., Hernandez, D., Ochner, C.N., Ungredda, T., Miller, R. and Kolbe, L. 2013. Supermarket discounts of low-energy density foods: effects on purchasing, food intake, and body weight. *Obesity (Silver Spring, Md.).* **21**(12), pp.E542-548.

Guan, X., Atlas, S.A. and Vadiveloo, M. 2018. Targeted retail coupons influence category-level food purchases over 2-years. *The International Journal of Behavioral Nutrition and Physical Activity Vol 15 2018, ArtID 111.* **15**(1), p111.

Hamilton, S., Mhurchu, C.N. and Priest, P. 2007. Food and nutrient availability in New Zealand: An analysis of supermarket sales data. *Public Health Nutrition.* **10**(12), pp.1448-1455.

Hansel, B., Roussel, R., Diguet, V., Deplaude, A., Chapman, M.J. and Bruckert, E. 2015. Relationships between consumption of alcoholic beverages and healthy foods: The French supermarket cohort of 196,000 subjects. *European Journal of Preventive Cardiology.* **22**(2), pp.215-222.

Hauser, M., Nussbeck, F.W. and Jonas, K. 2013. The impact of food-related values on food purchase behavior and the mediating role of attitudes: A Swiss study. *Psychology & Marketing.* **30**(9), pp.765-778.

Hobin, E., Bollinger, B., Sacco, J., Liebman, E., Vanderlee, L., Zuo, F., Rosella, L., L'Abbe, M., Manson, H. and Hammond, D. 2017. Consumers' response to an on-shelf nutrition labelling system in supermarkets: Evidence to inform policy and practice. *Milbank Quarterly.* **95**(3), pp.494-534.

Johansen, D., Friis, K., Skovenborg, E. and Grønbæk, M. 2006. Food buying habits of people who buy wine or beer: cross sectional study. *BMJ.* **332**(7540), pp.519-522.

Jones, E. 1997. An Analysis of Consumer Food Shopping Behavior Using Supermarket Scanner Data: Differences by Income and Location. *American Journal of Agricultural Economics.* **79**(5), pp.1437-1443.

Kroese, F.M., Marchiori, D.R. and de Ridder, D.T. 2016. Nudging healthy food choices: a field experiment at the train station. *Journal of public health (Oxford, England).* **38**(2), pp.e133-e137.

Le, H.N.D., Gold, L., Abbott, G., Crawford, D., McNaughton, S.A., Mhurchu, C.N., Pollard, C. and Ball, K. 2016. Economic evaluation of price discounts and skill-building strategies on purchase and consumption of healthy food and beverages: The SHELf randomized controlled trial. *Social Science & Medicine.* **159**, pp.83-91.

Martinez, O., Tagliaferro, B., Rodriguez, N., Athens, J., Abrams, C. and Elbel, B. 2018. EBT payment for online grocery orders: A mixed-methods study to understand its uptake among SNAP recipients and the barriers to and motivators for its use. *Journal of Nutrition Education and Behavior.* **50**(4), pp.396-402.

Mathios, A.D. 1998. The Importance of Nutrition Labeling and Health Claim Regulation on Product Choice: An Analysis of the Cooking Oils Market. *Agricultural and Resource Economics Review.* **27**(2), pp.159-168.

Mathios, A.D. 2000. The Impact of Mandatory Disclosure Laws on Product Choices: An Analysis of the Salad Dressing Market. *The Journal of Law & Economics.* **43**(2), pp.651-678.

Mhurchu, C.N., Blakely, T., Jiang, Y., Eyles, H.C. and Rodgers, A. 2010. Effects of price discounts and tailored nutrition education on supermarket purchases: A randomized controlled trial. *American Journal of Clinical Nutrition.* **91**(3), pp.736-747.

Mhurchu, C.N., Blakely, T., Wall, J., Rodgers, A., Jiang, Y. and Wilton, J. 2007. Strategies to promote healthier food purchases: A pilot supermarket intervention study. *Public Health Nutrition.* **10**(6), pp.608-615.

Moran, A.J., Khandpur, N., Polacsek, M., Thorndike, A.N., Franckle, R.L., Boulos, R., Sampson, S., Greene, J.C., Blue, D.G. and Rimm, E.B. 2019. Make It Fresh, for Less! A supermarket meal bundling and electronic reminder intervention to promote healthy purchases among families with children. *Journal of Nutrition Education and Behavior.* **51**(4), pp.400-408.

Mork, T., Grunert, K.G., Fenger, M., Juhl, H.J. and Tsalis, G. 2017. An analysis of the effects of a campaign supporting use of a health symbol on food sales and shopping behaviour of consumers. *BMC public health.* **17**(1), p239.

Närhinen, M., Berg, M.-A., Nissinen, A. and Puska, P. 1999. Supermarket sales data: a tool for measuring regional differences in dietary habits. *Public Health Nutrition.* **2**(3), pp.277-282.

Närhinen, M., Nissinen, A. and Puska, P. 1998. Sales data of a supermarket – a tool for monitoring nutrition interventions. *Public Health Nutrition.* **1**(2), pp.101-107.

Nevalainen, J., Erkkola, M., Saarijärvi, H., Näppilä, T. and Fogelholm, M. 2018. Large-scale loyalty card data in health research. *Digital health.* **4**, pp.2055207618816898-2055207618816898.

Payne, C.R., Niculescu, M., Just, D.R. and Kelly, M.P. 2015. Shopper marketing nutrition interventions: Social norms on grocery carts increase produce spending without increasing shopper budgets. *Preventive Medicine Reports.* **2**, pp.287-291.

Phipps, E.J., Kumanyika, S.K., Stites, S.D., Singletary, S.B., Cooblall, C. and DiSantis, K.I. 2014. Buying food on sale: a mixed methods study with shoppers at an urban supermarket, Philadelphia, Pennsylvania, 2010-2012. *Preventing chronic disease.* **11**, pE151.

Phipps, E.J., Stites, S.D., Wallace, S.L. and Braitman, L.E. 2013. Fresh fruit and vegetable purchases in an urban supermarket by low-income households. *Journal of Nutrition Education and Behavior.* **45**(2), pp.165-170.

Polacsek, M., Moran, A., Thorndike, A.N., Boulos, R., Franckle, R.L., Greene, J.C., Blue, D.J., Block, J.P. and Rimm, E.B. 2018. A Supermarket Double-Dollar Incentive Program Increases Purchases of Fresh Fruits and Vegetables Among Low-Income Families With Children: The Healthy Double Study. *Journal of nutrition education and behavior.* **50**(3), pp.217-228.

Radimer, K.L. and Harvey, P.W. 1998. Comparison of self-report of reduced fat and salt foods with sales and supply data. *European journal of clinical nutrition.* **52**(5), pp.380-382.

Reger, B., Wootan, M.G. and Booth-Butterfield, S. 1999. Using Mass Media to Promote Healthy Eating: A Community-Based Demonstration Project. *Preventive Medicine.* **29**(5), pp.414-421.

Reger, B., Wootan, M.G. and Booth-Butterfield, S. 2000. A comparison of different approaches to promote community-wide dietary change. *American journal of preventive medicine.* **18**(4), pp.271-275.

Reger, B., Wootan, M.G., Booth-Butterfield, S. and Smith, H. 1998. 1% or less: a community-based nutrition campaign. *Public Health Reports.* **113**(5), pp.410-419.

Revoredo-Giha, C., Lamprinopoulou, C., Toma, L., Leat, P.M.K., Kupiec-Teahan, B. and Cacciolatti, L. 2009. Bread prices, consumption and nutrition implications for Scotland: a regional analysis using supermarket scanner data. *Land Economy Working Paper Series - Scottish Agricultural College.* (48), pp.23-pp.

Schwartz, J., Mochon, D., Wyper, L., Maroba, J., Patel, D. and Ariely, D. 2014. Healthier by precommitment. *Psychological science.* **25**(2), pp.538-546.

Silver, L.D., Ng, S.W., Ryan-Ibarra, S., Taillie, L.S., Induni, M., Miles, D.R., Poti, J.M. and Popkin, B.M. 2017. Changes in prices, sales, consumer spending, and beverage consumption one year after a tax on sugar-sweetened beverages in Berkeley, California, US: A before-and-after study. *PLoS Medicine.* **14**(4), pe1002283.

Stead, M., MacKintosh, A.M., Findlay, A., Sparks, L., Anderson, A.S., Barton, K. and Eadie, D. 2017. Impact of a targeted direct marketing price promotion intervention (Buywell) on food-purchasing behaviour by low income consumers: a randomised controlled trial. *Journal of human nutrition and dietetics : the official journal of the British Dietetic Association.* **30**(4), pp.524-533.

Sturm, R., An, R.P., Segal, D. and Deepak, P. 2013. A cash-back rebate program for healthy food purchases in South Africa: results from scanner data. *American Journal of Preventive Medicine.* **44**(6), pp.567-572.

Sturm, R., Patel, D., Alexander, E. and Paramanund, J. 2016. Seasonal cycles in food purchases and changes in BMI among South Africans participating in a health promotion programme. *Public Health Nutrition.* **19**(15), pp.2838-2843.

Surkan, P.J., Tabrizi, M.J., Lee, R.M., Palmer, A.M. and Frick, K.D. 2016. Eat Right-Live Well! Supermarket intervention impact on sales of healthy foods in a low-income neighborhood. *Journal of Nutrition Education and Behavior.* **48**(2), pp.112-121.

Taylor, A., Wilson, F., Hendrie, G.A., Allman-Farinelli, M. and Noakes, M. 2015. Feasibility of a Healthy Trolley Index to assess dietary quality of the household food supply. *British Journal of Nutrition.* **114**(12), pp.2129-2137.

Toft, U., Winkler, L.L., Mikkelsen, B.E., Bloch, P. and Glumer, C. 2017. Discounts on fruit and vegetables combined with a space management intervention increased sales in supermarkets. *European journal of clinical nutrition.* **71**(4), pp.476-480.

Tran, L.T.T., Brewster, P.J., Chidambaram, V. and Hurdle, J.F. 2017. An innovative method for monitoring food quality and the healthfulness of consumers' grocery purchases. *Nutrients.* **9**(5), p457.

Uusitalo, L., Erkkola, M., Lintonen, T., Rahkonen, O. and Nevalainen, J. 2019. Alcohol expenditure in grocery stores and their associations with tobacco and food expenditures. *BMC Public Health.* **19**(787).

Van Gestel, L., Kroese, F. and De Ridder, D. 2018. Nudging at the checkout counter-A longitudinal study of the effect of a food repositioning nudge on healthy food choice. *Psychology & Health.* **33**(6), pp.800-809.

Vandenbroele, J., Slabbinck, H., Kerckhove, A.v. and Vermeir, I. 2018. Curbing portion size effects by adding smaller portions at the point of purchase. *Food Quality and Preference.* **64**, pp.82-87.

Walmsley, R., Jenkinson, D., Saunders, I., Howard, T. and Oyebode, O. 2018. Choice architecture modifies fruit and vegetable purchasing in a university campus grocery store: time series modelling of a natural experiment. *BMC Public Health.* **18**(1), p1149.

Winkler, L.L., Christensen, U., Glumer, C., Bloch, P., Mikkelsen, B.E., Wansink, B. and Toft, U. 2016. Substituting sugar confectionery with fruit and healthy snacks at checkout - a win-win strategy for consumers and food stores? a study on consumer attitudes and sales effects of a healthy supermarket intervention. *BMC public health.* **16**(1), p1184.

Table S5. NIH Quality Assessment Tool for Observational Cohort and Cross-Sectional Studies Risk of Bias assessment for included papers

| Author, year | 1. Question clear? | 2. Population clearly defined? | 3. >50% participate? | 4. Recruitment populations consistent? | 5. Sample size justified? | 6. Exposure assessed prior to outcome? | 7. Sufficient timeframe? | 8. Different exposure levels? | 9. Repeated exposure assessment? | 10. Valid outcomes? | 11. Outcome assessors blinded? | 12. Loss to follow up <20%? | 13. Confounders adjusted for? | Overall quality rating |
| --- | --- | --- | --- | --- | --- | --- | --- | --- | --- | --- | --- | --- | --- | --- |
| Alvarado et al. (2019) |  |  | ? | ? | X |  |  | X |  |  | NA | ? |  | Fair |
| An (2014) |  |  | ? |  | X |  |  |  |  |  | NA | ? | X | Fair |
| Andreyeva and Luedicke (2013) |  |  | ? |  | X |  |  | X |  |  | NA | ? |  | Fair |
| Andreyeva and Luedicke (2015) |  |  | ? |  | X |  |  |  |  |  | NA | ? |  | Fair |
| Andreyeva et al. (2014) |  |  | ? |  | X |  |  |  |  |  | NA | ? |  | Fair |
| Andreyeva et al. (2012) |  |  | ? |  | X |  |  |  |  |  | NA | ? |  | Fair |
| Andreyeva et al. (2013) |  |  | ? |  | X |  |  | X |  |  | NA | ? |  | Fair |
| Balasubramanian and Cole (2002) |  | ? | ? | ? | X |  |  |  |  |  | NA | ? | X | Poor |
| Ball, Kylie et al. (2016) |  |  | X |  | X |  |  | X |  |  |  |  | X | Fair |
| Ball, K. et al. (2015) |  |  |  | X |  |  |  |  |  |  |  |  |  | Good |
| Banerjee and Nayak (2018) |  |  | ? |  |  |  | X |  |  | X | ? |  |  | Poor |
| Bangia et al. (2017) |  |  |  |  | X |  |  |  | X |  | NA | X |  | Fair |
| Bartlett (2014) |  |  |  |  |  |  |  |  |  |  | ? | X |  | Good |
| Bernales-Korins et al. (2017) |  |  |  |  |  |  |  |  |  |  | ? | X |  | Good |
| Blakely et al. (2011) |  |  |  | X |  |  |  |  |  |  |  |  |  | Good |
| Brimblecombe et al. (2017) |  | X | ? |  |  |  |  |  |  |  | ? | ? | X | Fair |
| Brinkerhoff et al. (2011) |  | X | ? | ? | X |  | NA | NA | NA |  | NA | ? | NA | Poor |
| Brunello et al. (2012) |  | X | ? |  | X |  |  |  |  |  | X | ? |  | Poor |
| Brunello et al. (2014) |  | X | ? |  | X |  |  |  |  |  | X | ? |  | Poor |
| Chidambaram et al. (2013) | X | X | ? | ? | X |  |  |  |  | ? | NA |  | NA | Poor |
| Dunt et al. (1999) |  | X | ? | ? | X |  |  |  |  | ? | NA | ? | ? | Poor |
| Eyles, H. et al. (2010) |  |  |  |  |  |  |  | X | NA |  | NA |  | X | Fair |
| Eyles, Helen et al. (2010) |  |  | ? |  | X | NA | NA | NA | NA | ? | NA | ? | X | Poor |
| Ferguson et al. (2017) |  |  | ? | ? | X |  |  |  |  |  | NA | ? |  | Fair |
| Franckle et al. (2018) |  |  |  |  | X |  |  |  |  |  |  | X |  | Fair |
| Franckle et al. (2019) |  |  | ? |  | X |  |  | X |  |  | NA | ? |  | Fair |
| Frazao and Allshouse (1996) |  | X | ? | ? | X |  |  |  |  | X | NA | ? | X | Poor |
| Freedman and Connors (2010) |  |  | ? | ? | X |  | ? |  |  |  | NA | ? |  | Fair |
| Gamburzew et al. (2016) |  |  |  |  | X |  |  |  |  |  | NA | X |  | Good |
| Geliebter et al. (2013) |  |  |  |  |  |  |  |  |  |  | ? | X |  | Good |
| Guan et al. (2018) |  | X | ? | ? | X |  |  |  |  |  | NA | ? | X | Poor |
| Hamilton et al. (2007) |  |  |  |  | X | NA |  | NA | NA |  | NA |  | X | Fair |
| Hansel et al. (2015) |  |  | ? | ? | X |  |  |  |  |  | NA | ? |  | Fair |
| Hauser et al. (2013) |  |  | ? | ? | X |  |  |  | NA |  | NA | ? |  | Poor |
| Hobin et al. (2017) |  |  | ? | ? | X |  |  | X |  |  | NA | ? | X | Fair |
| Johansen et al. (2006) |  | X | ? | ? | X |  |  |  |  |  | NA | ? | X | Fair |
| Jones (1997) |  | ? | ? | ? | X |  |  |  |  |  | NA | ? | X | Poor |
| Kroese et al. (2016) |  | X | ? | ? | X |  |  |  | X |  | NA | ? | X | Poor |
| Le et al. (2016) |  |  |  | X |  |  |  |  |  |  |  |  |  | Good |
| Martinez et al. (2018) |  | X | X |  | X |  |  |  |  |  | NA | X | ? | Fair |
| Mathios (1998) |  |  | ? | ? | X |  |  |  |  |  | NA | ? |  | Fair |
| Mathios (2000) |  |  | ? | ? | X |  |  |  |  |  | NA | ? |  | Fair |
| Moran et al. (2019) |  |  |  |  | X |  |  |  |  | X | X |  | X | Poor |
| Mork et al. (2017) |  | X | ? | ? | X |  |  |  |  |  | NA | ? | ? | Poor |
| Närhinen et al. (1998) |  | X | ? | ? | X |  |  |  |  |  | NA | ? | ? | Fair |
| Närhinen et al. (1999) |  | ? | ? | ? | X |  |  |  |  |  | NA | ? | ? | Fair |
| Nevalainen et al. (2018) | X |  | X |  | X |  |  |  |  |  | NA |  |  | Fair |
| Ni Mhurchu et al. (2007) |  |  |  | X | X |  |  | NA | NA |  | ? |  | ? | Fair |
| Ni Mhurchu et al. (2010) |  |  |  | X |  |  |  |  |  |  |  |  |  | Good |
| Payne et al. (2015) |  |  | ? | ? | X |  |  |  |  |  | NA | ? | ? | Fair |
| Phipps, E. J. et al. (2014) |  |  | ? |  | X |  |  |  |  |  | NA | ? |  | Fair |
| Phipps, Etienne J. et al. (2013) |  |  | ? |  | X |  |  |  |  |  | NA | ? |  | Fair |
| Polacsek et al. (2018) |  |  |  |  | X |  |  |  |  |  |  |  |  | Good |
| Radimer and Harvey (1998) |  | X | X |  | X |  |  |  |  |  | NA | ? | X | Poor |
| Reger, B. et al. (2000) |  |  | ? |  | X |  |  |  |  |  | NA | X | ? | Fair |
| Reger, B. et al. (1998) |  | X | ? | ? | ? |  |  | X |  |  | NA |  |  | Fair |
| Reger, Bill et al. (1999) |  |  | ? | ? | X |  | ? | X |  |  | NA | ? | X | Poor |
| Revoredo-Giha et al. (2009) |  | X | ? | ? | X |  |  |  | NA |  | NA | ? | ? | Fair |
| Schwartz et al. (2014) |  |  |  |  | X |  |  |  |  |  | ? |  |  | Fair |
| Silver et al. (2017) |  |  | X |  |  |  |  |  |  |  | X | X |  | Fair |
| Stead et al. (2017) |  |  | ? |  | X |  |  |  |  |  | ? |  | ? | Fair |
| Sturm et al. (2013) |  |  |  |  | X |  |  |  |  |  | X | ? |  | Fair |
| Sturm et al. (2016) |  |  |  |  | X |  |  | NA |  |  | NA |  |  | Good |
| Surkan et al. (2016) |  | X | ? |  | X |  |  |  |  |  | NA | ? | ? | Fair |
| Taylor et al. (2015) |  |  | ? |  | X |  |  |  |  |  | NA |  |  | Fair |
| Toft et al. (2017) |  |  | ? | ? | X |  |  |  |  |  | X | ? | ? | Fair |
| Tran et al. (2017) | X | X | ? | ? | ? |  |  |  |  |  | NA | ? | NA | Poor |
| Uusitalo et al. (2019) |  |  |  | ? | X |  |  |  |  |  | NA | ? |  | Fair |
| Van Gestel et al. (2018) |  | X | ? | ? | X |  |  |  |  |  | NA | ? | X | Poor |
| Vandenbroele et al. (2018) |  | X | ? | ? | X |  |  |  |  |  | NA | ? |  | Fair |
| Walmsley et al. (2018) |  | X | ? | ? | X |  |  |  |  |  | NA | ? | X | Poor |
| Winkler et al. (2016) |  |  | ? | ? | X |  | ? |  |  |  | NA | ? | ? | Fair |

- = Yes, X = No, NA = Not Applicable, ? = Not Reported or Cannot Determine

### References S5.

Alvarado, M., Unwin, N., Sharp, S.J., Hambleton, I., Murphy, M.M., Samuels, T.A., Suhrcke, M. and Adams, J. 2019. Assessing the impact of the Barbados sugar-sweetened beverage tax on beverage sales: an observational study. *International Journal of Behavioral Nutrition & Physical Activity.* **16**(1), p13.

An, R. 2014. Eating better for less: Effectiveness of financial incentives in modifying dietary and grocery shopping behavior. *Dissertation Abstracts International: Section B: The Sciences and Engineering.* **74**(9-B(E)), pp.No-Specified.

Andreyeva, T. and Luedicke, J. 2013. Federal food package revisions: effects on purchases of whole-grain products. *American Journal of Preventive Medicine.* **45**(4), pp.422-429.

Andreyeva, T. and Luedicke, J. 2015. Incentivizing fruit and vegetable purchases among participants in the special supplemental nutrition program for women, infants, and children. *Public Health Nutrition.* **18**(1), pp.33-41.

Andreyeva, T., Luedicke, J., Henderson, K.E. and Schwartz, M.B. 2014. The positive effects of the revised milk and cheese allowances in the special supplemental nutrition program for women, infants, and children. *Journal of the Academy of Nutrition and Dietetics.* **114**(4), pp.622-630.

Andreyeva, T., Luedicke, J., Henderson, K.E. and Tripp, A.S. 2012. Grocery store beverage choices by participants in federal food assistance and nutrition programs. *American Journal of Preventive Medicine.* **43**(4), pp.411-418.

Andreyeva, T., Luedicke, J., Tripp, A.S. and Henderson, K.E. 2013. Effects of reduced juice allowances in food packages for the women, infants, and children program. *Pediatrics.* **131**(5), pp.919-927.

Balasubramanian, S.K. and Cole, C. 2002. Consumers' search and use of nutrition information: The challenge and promise of the Nutrition Labeling and Education Act. *Journal of Marketing.* **66**(3), pp.112-127.

Ball, K., McNaughton, S.A., Le, H.N., Abbott, G., Stephens, L.D. and Crawford, D.A. 2016. ShopSmart 4 Health: results of a randomized controlled trial of a behavioral intervention promoting fruit and vegetable consumption among socioeconomically disadvantaged women. *The American journal of clinical nutrition.* **104**(2), pp.436-445.

Ball, K., McNaughton, S.A., Le, H.N.D., Gold, L., Ni Mhurchu, C., Abbott, G., Pollard, C. and Crawford, D. 2015. Influence of price discounts and skill-building strategies on purchase and consumption of healthy food and beverages: Outcomes of the supermarket healthy eating for life randomized controlled trial. *American Journal of Clinical Nutrition.* **101**(5), pp.1055-1064.

Banerjee, T. and Nayak, A. 2018. Believe it or not: Health education works. *Obesity Research and Clinical Practice.* **12**(1), pp.116-124.

Bangia, D., Shaffner, D.W. and Palmer-Keenan, D.M. 2017. A Point-of-Purchase Intervention Using Grocery Store Tour Podcasts About Omega-3s Increases Long-Term Purchases of Omega-3-Rich Food Items. *Journal of nutrition education and behavior.* **49**(6), pp.475-480.

Bartlett, S., Klerman, J., Olsha, L., Logan, C., Blocklin, M., Beaurgard, M., Enver, A. 2014. *Evaluation of the Healthy Incentives Pilot (HIP) Final Report.*

Bernales-Korins, M., Ang, I.Y.H., Khan, S. and Geliebter, A. 2017. Psychosocial influences on fruit and vegetable intake following a NYC supermarket discount. *Obesity.* **25**(8), pp.1321-1328.

Blakely, T., Mhurchu, C.N., Jiang, Y., Matoe, L., Funaki-Tahifote, M., Eyles, H.C., Foster, R.H., McKenzie, S. and Rodgers, A. 2011. Do effects of price discounts and nutrition education on food purchases vary by ethnicity, income and education? Results from a randomised, controlled trial. *Journal of Epidemiology and Community Health.* **65**(10), pp.902-908.

Brimblecombe, J., Ferguson, M., Chatfield, M.D., Liberato, S.C., Gunther, A., Ball, K., Moodie, M., Miles, E., Magnus, A., Mhurchu, C.N., Leach, A.J., Bailie, R. and collaborative, S.R.r. 2017. Effect of a price discount and consumer education strategy on food and beverage purchases in remote Indigenous Australia: a stepped-wedge randomised controlled trial. *The Lancet. Public health.* **2**(2), pp.e82-e95.

Brinkerhoff, K.M., Brewster, P.J., Clark, E.B., Jordan, K.C., Cummins, M.R. and Hurdle, J.F. 2011. Linking Supermarket Sales Data To Nutritional Information: An Informatics Feasibility Study. *AMIA Annual Symposium Proceedings.* **2011**, pp.598-606.

Brunello, G., Paola, M.d. and Labartino, G. 2012. More apples less chips? The effect of school fruit schemes on the consumption of junk food. *IZA Discussion Papers - Forschungsinstitut zur Zukunft der Arbeit.* (6496), pp.23-pp.

Brunello, G., Paola, M.d. and Labartino, G. 2014. More apples fewer chips? The effect of school fruit schemes on the consumption of junk food. *Health Policy.* **118**(1), pp.114-126.

Chidambaram, V., Brewster, P.J., Jordan, K.C. and Hurdle, J.F. 2013. qDIET: toward an automated, self-sustaining knowledge base to facilitate linking point-of-sale grocery items to nutritional content. *AMIA ... Annual Symposium proceedings. AMIA Symposium.* **2013**, pp.224-233.

Dunt, D., Day, N. and Pirkis, J. 1999. Evaluation of a community-based health promotion program supporting public policy initiatives for a healthy diet. *Health Promotion International.* **14**(4), pp.317-327.

Eyles, H., Jiang, Y. and Ni Mhurchu, C. 2010. Use of household supermarket sales data to estimate nutrient intakes: a comparison with repeat 24-hour dietary recalls. *Journal of the American Dietetic Association.* **110**(1), pp.106-110.

Eyles, H., Rodgers, A. and Ni Mhurchu, C. 2010. Use of electronic sales data to tailor nutrition education resources for an ethnically diverse population. *Journal of Human Nutrition and Dietetics.* **23**(1), pp.38-47.

Ferguson, M., O'Dea, K., Holden, S., Miles, E. and Brimblecombe, J. 2017. Food and beverage price discounts to improve health in remote Aboriginal communities: mixed method evaluation of a natural experiment. *Australian and New Zealand journal of public health.* **41**(1), pp.32-37.

Franckle, R.L., Levy, D.E., Macias-Navarro, L., Rimm, E.B. and Thorndike, A.N. 2018. Traffic-light labels and financial incentives to reduce sugar-sweetened beverage purchases by low-income Latino families: a randomized controlled trial. *Public Health Nutrition.* **21**(8), pp.1426-1434.

Franckle, R.L., Thorndike, A.N., Moran, A.J., Hou, T., Blue, D., Greene, J.C., Bleich, S.N., Block, J.P., Polacsek, M. and Rimm, E.B. 2019. Supermarket Purchases Over the Supplemental Nutrition Assistance Program Benefit Month: A Comparison Between Participants and Nonparticipants. *American Journal of Preventive Medicine.* **57**(6), pp.800-807.

Frazao, E. and Allshouse, J.E. 1996. Size and growth of the nutritionally improved foods market. *Agriculture Information Bulletin - United States Department of Agriculture.* (723), pp.iv-pp.

Freedman, M.R. and Connors, R. 2010. Point-of-Purchase Nutrition Information Influences Food-Purchasing Behaviors of College Students: A Pilot Study. *Journal of the American Dietetic Association.* **110**(8), pp.1222-1226.

Gamburzew, A., Darcel, N., Gazan, R., Dubois, C., Maillot, M., Tome, D., Raffin, S. and Darmon, N. 2016. In-store marketing of inexpensive foods with good nutritional quality in disadvantaged neighborhoods: Increased awareness, understanding, and purchasing. *The International Journal of Behavioral Nutrition and Physical Activity.* **13**.

Geliebter, A., Ang, I.Y.H., Bernales-Korins, M., Hernandez, D., Ochner, C.N., Ungredda, T., Miller, R. and Kolbe, L. 2013. Supermarket discounts of low-energy density foods: effects on purchasing, food intake, and body weight. *Obesity (Silver Spring, Md.).* **21**(12), pp.E542-548.

Guan, X., Atlas, S.A. and Vadiveloo, M. 2018. Targeted retail coupons influence category-level food purchases over 2-years. *The International Journal of Behavioral Nutrition and Physical Activity Vol 15 2018, ArtID 111.* **15**(1), p111.

Hamilton, S., Mhurchu, C.N. and Priest, P. 2007. Food and nutrient availability in New Zealand: An analysis of supermarket sales data. *Public Health Nutrition.* **10**(12), pp.1448-1455.

Hansel, B., Roussel, R., Diguet, V., Deplaude, A., Chapman, M.J. and Bruckert, E. 2015. Relationships between consumption of alcoholic beverages and healthy foods: The French supermarket cohort of 196,000 subjects. *European Journal of Preventive Cardiology.* **22**(2), pp.215-222.

Hauser, M., Nussbeck, F.W. and Jonas, K. 2013. The impact of food-related values on food purchase behavior and the mediating role of attitudes: A Swiss study. *Psychology & Marketing.* **30**(9), pp.765-778.

Hobin, E., Bollinger, B., Sacco, J., Liebman, E., Vanderlee, L., Zuo, F., Rosella, L., L'Abbe, M., Manson, H. and Hammond, D. 2017. Consumers' response to an on-shelf nutrition labelling system in supermarkets: Evidence to inform policy and practice. *Milbank Quarterly.* **95**(3), pp.494-534.

Johansen, D., Friis, K., Skovenborg, E. and Grønbæk, M. 2006. Food buying habits of people who buy wine or beer: cross sectional study. *BMJ.* **332**(7540), pp.519-522.

Jones, E. 1997. An Analysis of Consumer Food Shopping Behavior Using Supermarket Scanner Data: Differences by Income and Location. *American Journal of Agricultural Economics.* **79**(5), pp.1437-1443.

Kroese, F.M., Marchiori, D.R. and de Ridder, D.T. 2016. Nudging healthy food choices: a field experiment at the train station. *Journal of public health (Oxford, England).* **38**(2), pp.e133-e137.

Le, H.N.D., Gold, L., Abbott, G., Crawford, D., McNaughton, S.A., Mhurchu, C.N., Pollard, C. and Ball, K. 2016. Economic evaluation of price discounts and skill-building strategies on purchase and consumption of healthy food and beverages: The SHELf randomized controlled trial. *Social Science & Medicine.* **159**, pp.83-91.

Martinez, O., Tagliaferro, B., Rodriguez, N., Athens, J., Abrams, C. and Elbel, B. 2018. EBT payment for online grocery orders: A mixed-methods study to understand its uptake among SNAP recipients and the barriers to and motivators for its use. *Journal of Nutrition Education and Behavior.* **50**(4), pp.396-402.

Mathios, A.D. 1998. The Importance of Nutrition Labeling and Health Claim Regulation on Product Choice: An Analysis of the Cooking Oils Market. *Agricultural and Resource Economics Review.* **27**(2), pp.159-168.

Mathios, A.D. 2000. The Impact of Mandatory Disclosure Laws on Product Choices: An Analysis of the Salad Dressing Market. *The Journal of Law & Economics.* **43**(2), pp.651-678.

Mhurchu, C.N., Blakely, T., Jiang, Y., Eyles, H.C. and Rodgers, A. 2010. Effects of price discounts and tailored nutrition education on supermarket purchases: A randomized controlled trial. *American Journal of Clinical Nutrition.* **91**(3), pp.736-747.

Mhurchu, C.N., Blakely, T., Wall, J., Rodgers, A., Jiang, Y. and Wilton, J. 2007. Strategies to promote healthier food purchases: A pilot supermarket intervention study. *Public Health Nutrition.* **10**(6), pp.608-615.

Moran, A.J., Khandpur, N., Polacsek, M., Thorndike, A.N., Franckle, R.L., Boulos, R., Sampson, S., Greene, J.C., Blue, D.G. and Rimm, E.B. 2019. Make It Fresh, for Less! A supermarket meal bundling and electronic reminder intervention to promote healthy purchases among families with children. *Journal of Nutrition Education and Behavior.* **51**(4), pp.400-408.

Mork, T., Grunert, K.G., Fenger, M., Juhl, H.J. and Tsalis, G. 2017. An analysis of the effects of a campaign supporting use of a health symbol on food sales and shopping behaviour of consumers. *BMC public health.* **17**(1), p239.

Närhinen, M., Berg, M.-A., Nissinen, A. and Puska, P. 1999. Supermarket sales data: a tool for measuring regional differences in dietary habits. *Public Health Nutrition.* **2**(3), pp.277-282.

Närhinen, M., Nissinen, A. and Puska, P. 1998. Sales data of a supermarket – a tool for monitoring nutrition interventions. *Public Health Nutrition.* **1**(2), pp.101-107.

Nevalainen, J., Erkkola, M., Saarijärvi, H., Näppilä, T. and Fogelholm, M. 2018. Large-scale loyalty card data in health research. *Digital health.* **4**, pp.2055207618816898-2055207618816898.

Payne, C.R., Niculescu, M., Just, D.R. and Kelly, M.P. 2015. Shopper marketing nutrition interventions: Social norms on grocery carts increase produce spending without increasing shopper budgets. *Preventive Medicine Reports.* **2**, pp.287-291.

Phipps, E.J., Kumanyika, S.K., Stites, S.D., Singletary, S.B., Cooblall, C. and DiSantis, K.I. 2014. Buying food on sale: a mixed methods study with shoppers at an urban supermarket, Philadelphia, Pennsylvania, 2010-2012. *Preventing chronic disease.* **11**, pE151.

Phipps, E.J., Stites, S.D., Wallace, S.L. and Braitman, L.E. 2013. Fresh fruit and vegetable purchases in an urban supermarket by low-income households. *Journal of Nutrition Education and Behavior.* **45**(2), pp.165-170.

Polacsek, M., Moran, A., Thorndike, A.N., Boulos, R., Franckle, R.L., Greene, J.C., Blue, D.J., Block, J.P. and Rimm, E.B. 2018. A Supermarket Double-Dollar Incentive Program Increases Purchases of Fresh Fruits and Vegetables Among Low-Income Families With Children: The Healthy Double Study. *Journal of nutrition education and behavior.* **50**(3), pp.217-228.

Radimer, K.L. and Harvey, P.W. 1998. Comparison of self-report of reduced fat and salt foods with sales and supply data. *European journal of clinical nutrition.* **52**(5), pp.380-382.

Reger, B., Wootan, M.G. and Booth-Butterfield, S. 1999. Using Mass Media to Promote Healthy Eating: A Community-Based Demonstration Project. *Preventive Medicine.* **29**(5), pp.414-421.

Reger, B., Wootan, M.G. and Booth-Butterfield, S. 2000. A comparison of different approaches to promote community-wide dietary change. *American journal of preventive medicine.* **18**(4), pp.271-275.

Reger, B., Wootan, M.G., Booth-Butterfield, S. and Smith, H. 1998. 1% or less: a community-based nutrition campaign. *Public Health Reports.* **113**(5), pp.410-419.

Revoredo-Giha, C., Lamprinopoulou, C., Toma, L., Leat, P.M.K., Kupiec-Teahan, B. and Cacciolatti, L. 2009. Bread prices, consumption and nutrition implications for Scotland: a regional analysis using supermarket scanner data. *Land Economy Working Paper Series - Scottish Agricultural College.* (48), pp.23-pp.

Schwartz, J., Mochon, D., Wyper, L., Maroba, J., Patel, D. and Ariely, D. 2014. Healthier by precommitment. *Psychological science.* **25**(2), pp.538-546.

Silver, L.D., Ng, S.W., Ryan-Ibarra, S., Taillie, L.S., Induni, M., Miles, D.R., Poti, J.M. and Popkin, B.M. 2017. Changes in prices, sales, consumer spending, and beverage consumption one year after a tax on sugar-sweetened beverages in Berkeley, California, US: A before-and-after study. *PLoS Medicine.* **14**(4), pe1002283.

Stead, M., MacKintosh, A.M., Findlay, A., Sparks, L., Anderson, A.S., Barton, K. and Eadie, D. 2017. Impact of a targeted direct marketing price promotion intervention (Buywell) on food-purchasing behaviour by low income consumers: a randomised controlled trial. *Journal of human nutrition and dietetics : the official journal of the British Dietetic Association.* **30**(4), pp.524-533.

Sturm, R., An, R.P., Segal, D. and Deepak, P. 2013. A cash-back rebate program for healthy food purchases in South Africa: results from scanner data. *American Journal of Preventive Medicine.* **44**(6), pp.567-572.

Sturm, R., Patel, D., Alexander, E. and Paramanund, J. 2016. Seasonal cycles in food purchases and changes in BMI among South Africans participating in a health promotion programme. *Public Health Nutrition.* **19**(15), pp.2838-2843.

Surkan, P.J., Tabrizi, M.J., Lee, R.M., Palmer, A.M. and Frick, K.D. 2016. Eat Right-Live Well! Supermarket intervention impact on sales of healthy foods in a low-income neighborhood. *Journal of Nutrition Education and Behavior.* **48**(2), pp.112-121.

Taylor, A., Wilson, F., Hendrie, G.A., Allman-Farinelli, M. and Noakes, M. 2015. Feasibility of a Healthy Trolley Index to assess dietary quality of the household food supply. *British Journal of Nutrition.* **114**(12), pp.2129-2137.

Toft, U., Winkler, L.L., Mikkelsen, B.E., Bloch, P. and Glumer, C. 2017. Discounts on fruit and vegetables combined with a space management intervention increased sales in supermarkets. *European journal of clinical nutrition.* **71**(4), pp.476-480.

Tran, L.T.T., Brewster, P.J., Chidambaram, V. and Hurdle, J.F. 2017. An innovative method for monitoring food quality and the healthfulness of consumers' grocery purchases. *Nutrients.* **9**(5), p457.

Uusitalo, L., Erkkola, M., Lintonen, T., Rahkonen, O. and Nevalainen, J. 2019. Alcohol expenditure in grocery stores and their associations with tobacco and food expenditures. *BMC Public Health.* **19**(787).

Van Gestel, L., Kroese, F. and De Ridder, D. 2018. Nudging at the checkout counter-A longitudinal study of the effect of a food repositioning nudge on healthy food choice. *Psychology & Health.* **33**(6), pp.800-809.

Vandenbroele, J., Slabbinck, H., Kerckhove, A.v. and Vermeir, I. 2018. Curbing portion size effects by adding smaller portions at the point of purchase. *Food Quality and Preference.* **64**, pp.82-87.

Walmsley, R., Jenkinson, D., Saunders, I., Howard, T. and Oyebode, O. 2018. Choice architecture modifies fruit and vegetable purchasing in a university campus grocery store: time series modelling of a natural experiment. *BMC Public Health.* **18**(1), p1149.

Winkler, L.L., Christensen, U., Glumer, C., Bloch, P., Mikkelsen, B.E., Wansink, B. and Toft, U. 2016. Substituting sugar confectionery with fruit and healthy snacks at checkout - a win-win strategy for consumers and food stores? a study on consumer attitudes and sales effects of a healthy supermarket intervention. *BMC public health.* **16**(1), p1184.
